# Association of circulating metabolites with healthy diet and risk of cardiovascular disease: analysis of two cohort studies

**DOI:** 10.1038/s41598-018-26441-1

**Published:** 2018-06-05

**Authors:** Tasnime Akbaraly, Peter Würtz, Archana Singh-Manoux, Martin J. Shipley, Rita Haapakoski, Maili Lehto, Catherine Desrumaux, Mika Kähönen, Terho Lehtimäki, Vera Mikkilä, Aroon Hingorani, Steve E. Humphries, Antti J. Kangas, Pasi Soininen, Olli Raitakari, Mika Ala-Korpela, Mika Kivimäki

**Affiliations:** 1 Inserm U 1198, Montpellier, F-34000 France; 2 Department of Epidemiology and Public Health, London, University College London, London, United Kingdom; 3 Department of Psychiatry & Autism Resources Centre, Hospital and University Research Center of Montpellier- CHRU-, Montpellier, F-34000 France; 4 Computational Medicine, Faculty of Medicine, University of Oulu and Biocenter Oulu, Oulu, Finland; 5 INSERM, U1018, Centre for Research in Epidemiology and Population Health, Hôpital Paul Brousse, Villejuif, France; 6 Department of Clinical Physiology, University of Tampere School of Medicine and Tampere University Hospital, Tampere, Finland; 7 Department of Clinical Chemistry, Fimlab Laboratories and University of Tampere School of Medicine, Tampere, Finland; 8 Research Centre of Applied and Preventive Cardiovascular Medicine, University of Turku, Turku, Finland; 9 Centre for Cardiovascular Genetics, British Heart Foundation Laboratories, Institute of Cardiovascular Sciences, University College London, London, United Kingdom; 10 NMR Metabolomics Laboratory, School of Pharmacy, University of Eastern Finland, Kuopio, Finland; 11 Department of Clinical Physiology and Nuclear Medicine, Turku University Hospital, Turku, Finland; 12 Medical Research Council Integrative Epidemiology Unit at the University of Bristol, Bristol, United Kingdom; 13 Population Health Science, Bristol Medical School, University of Bristol, Bristol, UK; 14 Systems Epidemiology, Baker Heart and Diabetes Institute, Melbourne, VIC Australia; 15 Department of Epidemiology and Preventive Medicine, School of Public Health and Preventive Medicine, Faculty of Medicine, Nursing and Health Sciences, The Alfred Hospital, Monash University, Melbourne, VIC Australia; 16 Clinicum, Faculty of Medicine, University of Helsinki, Helsinki, Finland; 17 University Montpellier, Montpellier, F-34000 France; 18 EPHE, Paris, France

## Abstract

Diet may modify metabolomic profiles towards higher or lower cardiovascular disease (CVD) risk. We aimed to identify metabolite profiles associated with high adherence to dietary recommendations - the Alternative Healthy Eating Index (AHEI) - and the extent to which metabolites associated with AHEI also predict incident CVD. Relations between AHEI score and 80 circulating lipids and metabolites, quantified by nuclear magnetic resonance metabolomics, were examined using linear regression models in the Whitehall II study (n = 4824, 55.9 ± 6.1 years, 28.0% women) and were replicated in the Cardiovascular Risk in Young Finns Study (n = 1716, 37.7 ± 5.0 years, 56.3% women). We used Cox models to study associations between metabolites and incident CVD over the 15.8-year follow-up in the Whitehall II study. After adjustment for confounders, higher AHEI score (indicating healthier diet) was associated with higher degree of unsaturation of fatty acids (FA) and higher ratios of polyunsaturated FA, omega-3 and docosahexaenoic acid relative to total FA in both Whitehall II and Young Finns studies. A concordance of associations of metabolites with higher AHEI score and lower CVD risk was observed in Whitehall II. Adherence to healthy diet seems to be associated with specific FA that reduce risk of CVD.

## Introduction

The benefits of healthy diet are supported by nutritional epidemiological studies on coronary heart diseases^1^, respiratory diseases^2^ and healthy old-age phenotypes^3^. Recent advancements of high-throughput metabolite profiling in large epidemiological studies allow the determination of metabolites predicting the risk for cardiometabolic diseases, providing insights into the molecular mechanisms underlying age-related diseases, such as cardiovascular diseases (CVD)^4^. It has been hypothesized that metabolites are very responsive to dietary exposure as diet is an important source of metabolite variation and also induces metabolic response.

Few studies have examined the association between overall diet and metabolites and a majority of investigations assessed metabolites via mass spectrometry methods. In the EPIC-Potsdam cohort study of 2380 adults, for example, dietary patterns were derived through reduced rank regression methods to explain the maximum variations of metabolites^5^ and a weak association between habitual diet and serum metabolites was observed. In a subsample of 1977 participants of the ARIC study, amongst the 336 metabolites assessed, dietary pattern “sugar-rich food and beverages” was associated with 7 unsaturated long-chain fatty acids, five 2-hydroxybutyrate–related metabolites, two sex steroids, five γ-glutamyl dipeptides, and four metabolites in other pathways^6^ and in the Women’s Health Initiative study, Prudent dietary pattern was associated with 85 metabolites (mostly lipids)^7^. Another study, carried out on 502 participants from the Prostate, Lung Colorectal and Ovarian Cancer Screening Trial, examined the correlations between 412 metabolites, food groups and the Healthy Eating Index score^8^. The authors reported that 39 metabolites were associated with 13 dietary groups and concluded that the metabolomic approach might be useful in identifying biomarkers reflecting the effect of nutrition intakes on human metabolism. In agreement with this, results from a study assessing lipoprotein particle subclasses profile via Nuclear Magnetic Resonance (NMR) in 663 adults showed associations between specific dietary patterns (“fish” and “junked food” pattern) and lipoprotein subclasses^9^. Identifying robust associations between dietary habits and metabolites may offer the possibility to better understand pathways by which overall diet mediates protection against chronic diseases, such as CVD, but none of these studies examined this issue.

In this study, we sought to identify metabolites associated with adherence to a healthy diet and to determine the extent to which these metabolites are also related to reduced risk of CVD. To do so, we assessed adherence to dietary guidelines in a large cohort of British middle-aged men and women from the Whitehall II study^10^ using the Alternative Healthy Eating Index (AHEI) – a dietary index whose high scores have been shown to be associated with reduced risk of CVD morbidity^11^ and mortality^12^. We examined associations of healthy diet with metabolites quantified using a serum NMR metabolomics and replicated the results in an independent cohort, the Cardiovascular Risk in Young Finns Study^13^. We then determined the extent to which metabolites associated with AHEI were also associated with the risk of developing CVD over 15.8 years of follow-up in the Whitehall II study.

## Results

### Participant characteristics

A total of 4824 participants from the Whitehall II study were included in the discovery analysis. Characteristics are described in Table 1. Mean concentration of the 80 metabolites are detailed in Supplementary Material-Table A. The mean (±SD) score of AHEI was 50.7 ± 9.8 points. Compared to the 3034 participants who attended the 1997/99 examination but were not included in the present analysis, those included were more likely to be men, white, with high socio-economic status, to practice physical activity and to report higher total energy intake. Participants included were also less likely to be smoker, to use antihypertensive or lipids lowering drugs and showed lower concentrations of triglycerides and lower diastolic blood pressure. No significant difference in AHEI score was observed (Supplementary Material- Table B). Regarding participants included in the Young Finns Study (replication cohort), the latter were younger and showed lower means of AHEI compared to participants included in Whitehall II study (Table 1).

**Table 1:** Characteristics of Whitehall II participants and Young Finns Study participants.

Characteristics		Whitehall II		Young Finns Study	
		N	% or mean (SD)	N	mean ± SD or ρ*
Sex	Men	3483	72.2	966	56.3
	Women	1341	27.8	750	43.7
Age, years		4824	55.9 (6.1)	1716	37.7 (5.0)
Ethnicity	White	4541	93.9	1716	1716 (100.0)
	South Asian	183	3.9	/	/
	Black	100	2.2	/	/
Smoking habits	Non	2490	52.1	886	51.6
	Former	1864	39.1	421	24.5
	Current	470	8.9	409	23.8
Physical activity MET unit /hours/week		4824	15.6 (14.8)	1716	19.6 (21.5)
Total score in AHEI, points		4824	50.7 (9.8)	1716	46.3 (8.0)
Total energy intake, kcal/day		4824	2233 (683)	1716	2392 (800)
Prevalent type 2 diabetes	No	4528	93.9	1665	97.0
	Yes	296	6.1	51	3.0
Systolic blood pressure, mmHg		4824	123.1 (16.5)	1716	120.3 (14.3)
Diastolic blood pressure, mmHg		4824	77.4 (10.5)	1714	75.4 (11.4)
Use of antihypertensive treatment	No	4 229	87.4	1604	93.5
	Yes	607	12.6	112	6.5
Triglycerides, mmol/L		4823	1.35 (0.86)	1714	1.38 (0.90)
HDL-cholesterol, mmol/L		4298	1.46 (0.39)	1708	1.34 (0.32)
Use of lipids lowering drugs	No	4669	96.9	1686	98.2
	Yes	155	3.1	30	1.7
Body mass index, kg/m²		4175	26.0 (3.9)	1695	25.8 (4.7)

### Association between AHEI score and metabolites in the Whitehall II study

**Figure 1:**
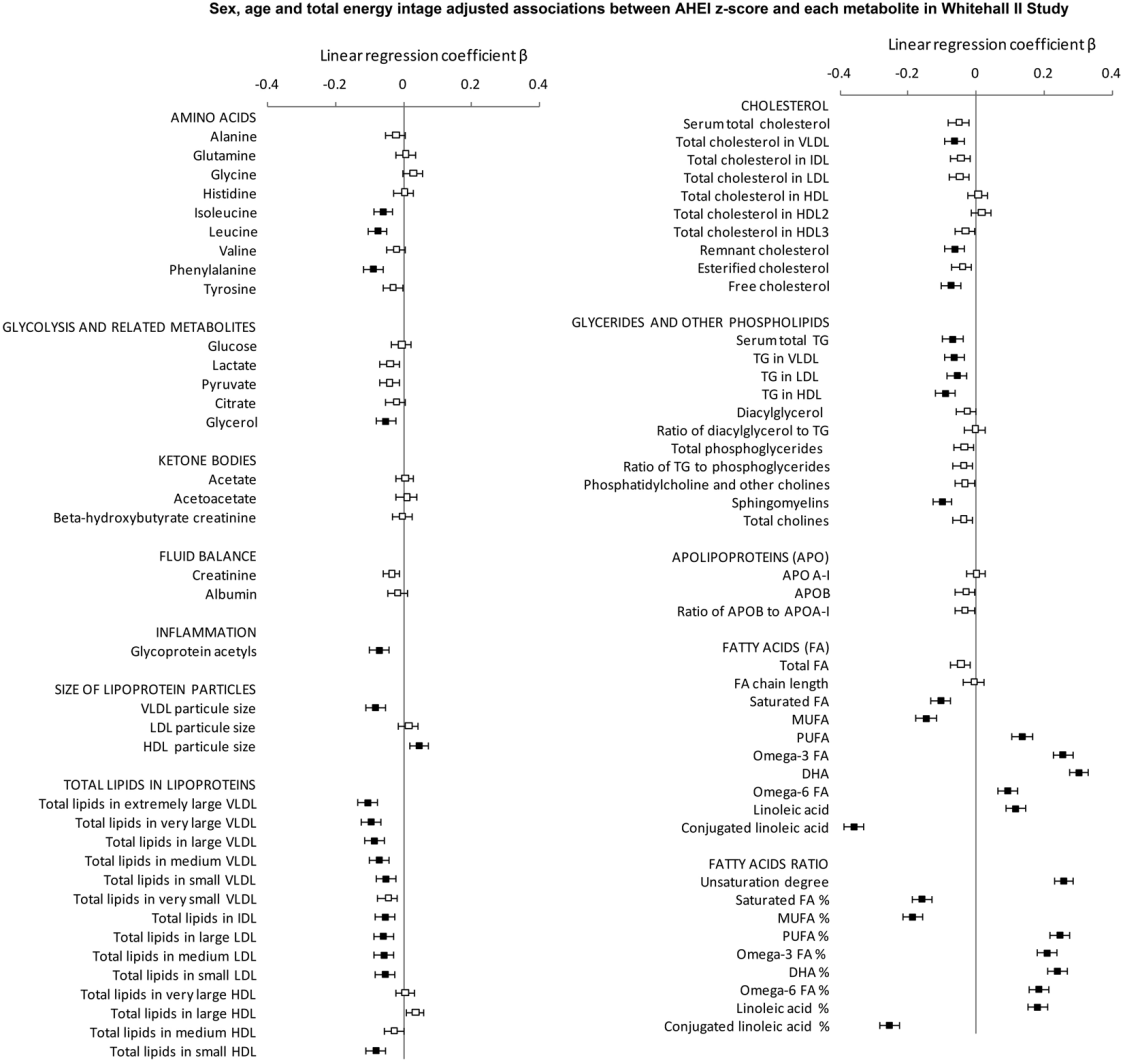
Age-, sex- and energy intake-adjusted associations between AHEI z-score and metabolites in Whitehall II study. Results are expressed as regression coefficients accompanied with their 95% confidence interval for one standard deviation increment in AHEI diet score. To facilitate comparison, metabolites were square root transformed and standardized to z-scores (mean = 0, SD = 1).  P ≥ 0.0006;  P < 0.0006.

Results of the associations between AHEI z-score and 80 metabolites in Whitehall II study are shown in Fig. 1, (estimates and p values are available in Supplementary Material-Table C). Good adherence to healthy dietary recommendations, as assessed by higher AHEI score, was associated with lower circulating concentrations of specific amino acids (isoleucine, leucine and phenylalanine) and of metabolites related to gluconeogenesis (mainly glycerol) as well as lower chronic inflammation (assessed by glycoprotein acetyls) after accounting for Bonferroni correction for 80 tests. Adherence to healthy diet was also associated with a smaller average size of VLDL particles and larger average size of HDL particles. Regarding lipids in different lipoproteins subclasses, participants with higher AHEI score showed lower concentration of lipids in VLDL, IDL and LDL particles (from large to small) and with lipids in small HDL particles. AHEI score was also inversely associated with concentrations of cholesterol in VLDL, cholesterol not contained in HDL nor LDL (remnant cholesterol) and with free cholesterol. Higher AHEI score was associated with lower triglycerides concentrations in all lipid subfractions and lower circulating sphingomyelins.

The strongest associations between metabolites and AHEI score were observed for fatty acid measures, especially for monounsaturated and conjugated linoleic acids for which linear regression coefficients were three times higher than for other metabolites on average (Supplementary Material-Table B). Regarding fatty acids, high AHEI score was associated with lower concentrations of saturated and monounsaturated fatty acids. Conversely, participants with higher AHEI score displayed higher concentrations of polyunsaturated fatty acids, including omega-3 (docosahexaenoic acid especially) and omega-6 (linoleic acids) but lower concentrations of conjugated linoleic acids. Analyses for ratios of each fatty acids category relative to total fatty acid concentrations confirmed the association between AHEI score and fatty acids and tended to display even stronger associations. All analyses were repeated by replacing AHEI by AHEI 2010 and similar trends were observed. Results are detailed in Supplementary Material-Table D.

The metabolites associations were only modestly attenuated (30.7% on average) after further adjustment for ethnicity, physical activity, smoking habits and cardiovascular risk factors (including type 2 diabetes, diastolic and systolic blood pressure, use of antihypertensive drugs and use of lipid-lowering drugs). All but one remained statistically significant (Table 2). An additional model in which body mass index (BMI) was added as covariate was performed. Analyses, carried out on the 4175 participants with available data on BMI, showed similar results (Supplementary Material-Table E).

**Table 2:** Results of multivariable adjusted linear regression models of the association between AHEI z-score and the 42 selected metabolites in the Whitehall II study and in Young Finns Study.

	Multivariable-adjusted Model*					
	Whitehall II (N = 4699)			Young Finns Study (N = 1625)		
	Beta	95% CI	p	Beta	95% CI	p
**Amino Acids**						
Isoleucine	−0.044	−0.072 to −0.017	0.002	0.011	−0.034 to 0.056	0.64
Leucine	−0.061	−0.088 to −0.034	1*10^−5^	0.010	−0.035 to 0.055	0.65
Phenylalanine	−0.074	−0.104 to −0.044	1*10^−6^	−0.038	−0.090 to 0.014	0.15
**Glycolysis related metabolites**						
Glycerol	−0.044	−0.074 to −0.014	0.004	−0.009	−0.060 to 0.042	0.73
**Inflammation**						
Glycoprotein acetyls	−0.035	−0.064 to −0.006	0.02	−0.012	−0.063 to 0.039	0.64
**Size of lipoprotein particles (Mean diameters)**						
VLDL particle size	−0.048	−0.076 to −0.019	0.0009	−0.003	−0.051 to 0.046	0.91
HDL particle size	0.033	0.006 to 0.061	0.02	−0.005	−0.051 to 0.040	0.82
**Total lipid concentrations in lipoprotein subclasses**						
Total lipids in chylomicrons and extremely large VLDL	−0.073	−0.102 to −0.044	8*10^−7^	−0.033	−0.082 to 0.016	0.19
Total lipids in very large VLDL	−0.061	−0.089 to −0.032	4*10^−5^	−0.022	−0.070 to 0.027	0.38
Total lipids in large VLDL	−0.049	−0.078 to −0.021	0.0007	−0.006	−0.054 to 0.042	0.81
Total lipids in medium VLDL	−0.036	−0.067 to −0.008	0.01	0.001	−0.048 to 0.048	0.99
Total lipids in small VLDL	−0.019	−0.048 to 0.010	0.21	0.017	−0.031 to 0.065	0.49
Total lipids in IDL	−0.039	−0.069 to −0.010	0.01	−0.032	−0.084 to 0.021	0.24
Total lipids in large LDL	−0.045	−0.075 to −0.015	0.003	−0.032	−0.084 to 0.021	0.24
Total lipids in medium LDL	−0.044	−0.074 to −0.014	0.004	−0.028	−0.080 to 0.024	0.29
Total lipids in small LDL	−0.039	−0.069 to −0.009	0.01	−0.028	−0.080 to 0.023	0.28
Total lipids in small HDL	−0.054	−0.083 to −0.024	0.0003	0.001	−0.052 to 0.054	0.96
**Cholesterol**						
Cholesterol in VLDL	−0.036	−0.065 to −0.006	0.02	−0.006	−0.055 to 0.043	0.81
Remnant cholesterol (non−HDL, non−LDL −cholesterol)	−0.041	−0.071 to −0.011	0.007	−0.021	−0.072 to 0.029	0.41
Free cholesterol	−0.055	−0.084 to −0.026	0.0002	−0.035	−0.089 to 0.018	0.19
**Glycerides and other Phospholipids**						
Serum total TG	−0.034	−0.063 to −0.005	0.02	−0.002	−0.050 to 0.047	0.95
TG in VLDL	−0.031	−0.061 to −0.003	0.04	0.001	−0.046 to 0.049	0.95
TG in LDL	−0.031	−0.060 to −0.002	0.04	−0.012	−0.064 to 0.041	0.66
TG in HDL	−0.060	−0.090 to −0.031	5*10^−5^	−0.017	−0.070 to 0.036	0.53
Sphingomyelins	−0.081	−0.110 to −0.053	2*10^−8^	−0.022	−0.074 to 0.031	0.42
**Fatty Acids (FA)**						
Saturated FA	−0.056	−0.086 to −0.027	0.0002	−0.029	−0.080 to 0.022	0.27
Monounsaturated FA	−0.069	−0.098 to −0.040	2*10^−6^	−0.022	−0.072 to 0.028	0.39
Polyunsaturated FA	0.076	0.047 to 0.106	4*10^−7^	0.028	−0.025 to 0.080	0.30
Omega-3 FA	0.140	0.111 to 0.169	1*10^−20^	0.097	0.045 to 0.149	0.0003
docosahexaenoic acid	0.176	0.147 to 0.205	1*10^−32^	0.096	0.044 to 0.149	0.0003
Omega-6 FA	0.054	0.024 to 0.083	0.0004	0.009	−0.043 to 0.062	0.72
linoleic acid	0.076	0.046 to 0.105	6*10^−7^	0.004	−0.049 to 0.056	0.89
Conjugated linoleic acid	−0.198	−0.227 to −0.169	2*10^−40^	NA	NA	
**Fatty acids ratios, relative to total fatty acids**						
Estimated degree of unsaturation	0.210	0.183 to 0.238	1*10^−48^	0.116	0.066 to 0.166	5*10^−6^
Ratio of saturated FA to total FA (%)	−0.164	−0.194 to −0.134	4*10^−27^	−0.089	−0.141 to −0.037	0.0008
Ratio of monounsaturated FA to total FA (%)	−0.124	−0.152 to −0.095	2*10^−17^	−0.034	−0.082 to 0.015	0.17
Ratio of polyunsaturated FA to total FA (%)	0.194	0.162 to 0.219	2*10^−41^	0.082	0.033 to 0.131	0.0011
Ratio of omega-3 FA to total FA (%)	0.190	0.162 to 0.219	1*10^−37^	0.142	0.091 to 0.193	5*10^−8^
Ratio of docosahexaenoic acid to total FA (%)	0.220	0.192 to 0.249	9*10^−52^	0.139	0.090 to 0.189	4*10^−8^
Ratio of omega-6 FA to total FA (%)	0.137	0.109 to 0.166	6*10^−21^	0.044	−0.006 to 0.094	0.08
Ratio of linoleic acid to total FA (%)	0.145	0.116 to 0.174	1*10^−22^	0.029	−0.022 to 0.080	0.26
Ratio of conjugated linoleic acid to total FA (%)	−0.226	−0.255 to −0.197	7*10^−52^	NA	NA	/

*Multivariable adjusted model: adjusted for age, sex, total energy intake, ethnicity, smoking habits, physical activity, type 2 diabetes, diastolic and systolic blood pressure, use of antihypertensive drugs and use of lipid-lowering drugs. Results were expressed as linear regression coefficients accompanied with their 95% confidence interval. Analyses were carried out on participants for which all metabolites measurement were available.

Because both dietary changes and a modification of circulating metabolites are expected in participants with prevalent CVD, cancer or longstanding illness, we performed sensitivity analyses to assess the extent by which the AHEI-metabolites associations reported here might be explained by these diseases. Analyses repeated after excluding participants (1) reporting history of cardiovascular diseases (2) with a diagnosis of cancer and (3) reporting a longstanding illness indicate that the associations reported were not explained by these chronic diseases (Supplemental Material-Table F).

### Replication analysis in the Young Finns study

**Figure 2:**
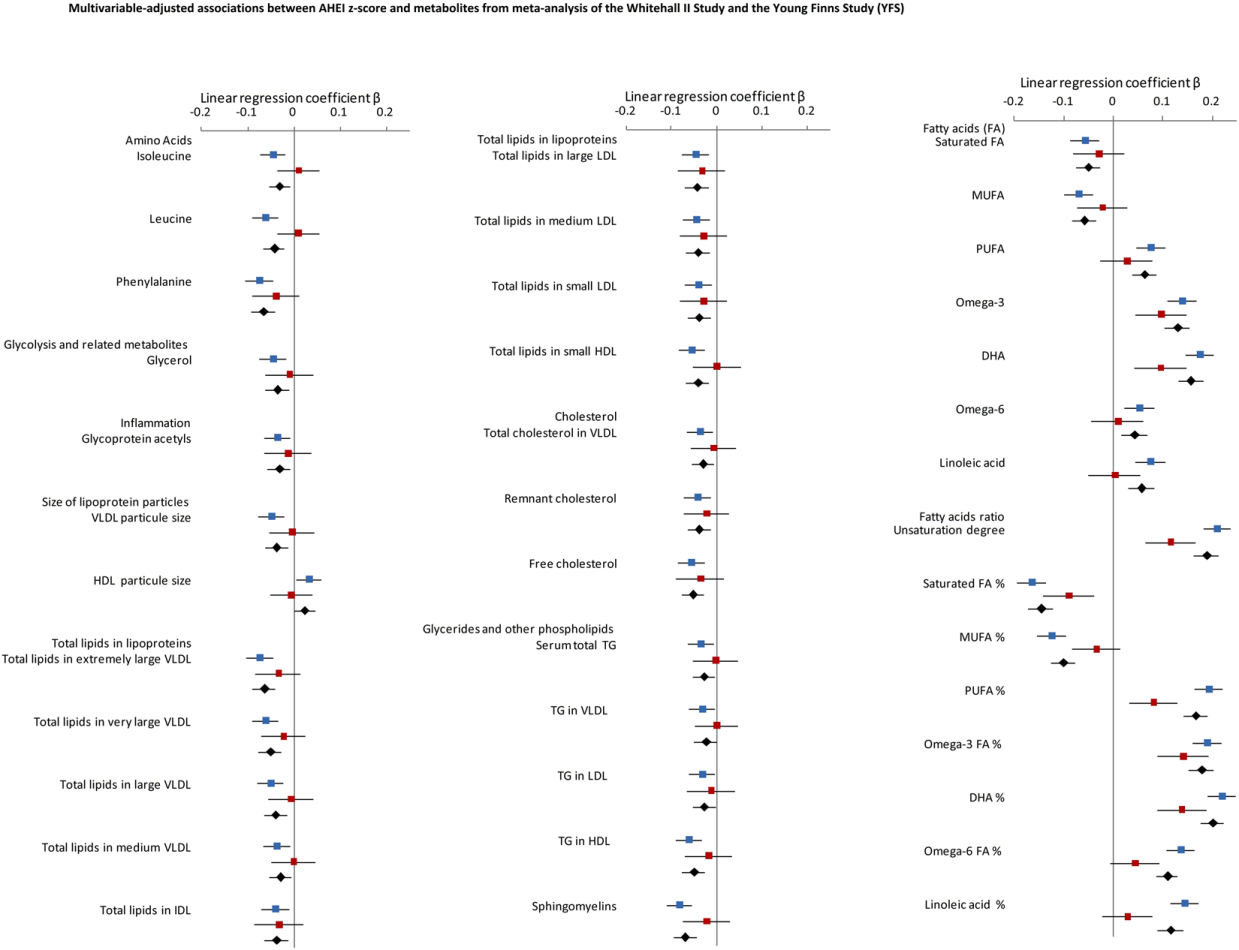
Multivariable-adjusted associations between AHEI z-score and metabolites from meta-analysis of the Whitehall II Study and the Young Finns Study (YFS).  Whitehall II study;  YFS;  Meta-analysis. Linear regression models were adjusted for age, sex, total energy intake, ethnicity, smoking habits, physical activity, type 2 diabetes, diastolic and systolic blood pressure, use of antihypertensive drugs and use of lipid-lowering drugs. Results are expressed as linear regression coefficients accompanied with their 95% confidence interval. To facilitate comparison, metabolites were first square root transformed and then standardized to z-scores (mean = 0, SD = 1).

Analyses of the associations between the 41 metabolites (those significantly associated with AHEI score in multivariate model performed in Whitehall II study) and AHEI z-score were repeated in the Young Finns Study whose sample effective was about the third of the Whitehall II effective. Mean concentration of these 41 metabolites in Young Finns are listed in Supplemental Material-Table G. The replication analyses and meta-analyses, displayed in Table 2 and Supplementary Material-Table H respectively and illustrated in Fig. 2 showed that 38 of the 41 diet-metabolites associations were directionally concordant. The only deviating measures are branched amino acids, glycerol and size of HDL particle.

Of the 41 metabolites assessed, AHEI score was significantly associated with two fatty acids and 5 fatty acids ratio (Fig. 2 and Supplementary Table H) confirming the strong associations between good adherence to healthy diet and higher concentrations of omega-3 and docosahexaenoic acid, higher ratios of all polyunsaturated fatty acids ratio (including omega-3, omega-6) and lower ratios of saturated and monounsaturated fatty acids relative to total fatty acids. Regarding the other metabolites, even if the direction of most of associations was similar as observed in Whitehall II, the associations were weaker and did not reach statistical significance in the Young Finns study with much smaller sample size than in Whitehall II.

### Metabolites associated with AHEI and predicting cardiovascular disease

We assessed the extent to which each of the 41 metabolites associated with diet score also predicted CVD events. Of the 5481 Whitehall II participants, 697 developed CVD over the 15.8 years of follow-up. Results are presented in Table 3. Metabolites found to significantly predict CVD risk consisted of amino acids, glycoprotein acetyls, size of lipoprotein particule size, total lipids in lipoproteins (except those in IDL and in small HDL), total cholesterol in VLDL particles and triglycerides. Amongst fatty acids, significant association were found for saturated fatty acids and monounsaturated fatty acids. Degree of unsaturation was inversely associated with CVD risk. When ratio of fatty acids categories relative to total fatty acids concentration was considered, significant associations were observed for mono- and poly-unsaturated fatty acids, omega-3 and docosahexaenoic acid, with higher ratio of monounsaturated fatty acids increasing the risk of CVD risk, and higher ratio of polyunsaturated fatty acids, omega-3 and docosahexaenoic acid decreasing CVD risk. Other fatty acids ratios were not found significantly associated with CVD risk.

**Table 3:** Association between baseline metabolites and incident cardiovascular disease over 15.8 years of follow-up in the Whitehall II study.

	Association with CVD risk (total N = 5481; N incident cases = 697)		
	Hazard Ratio*	95% CI	p
**Amino Acids**			
Isoleucine	1.14	1.06 to 1.24	0.001
Leucine	1.13	1.04 to 1.22	0.003
Phenylalanine	1.14	1.07 to 1.23	0.000
**Glycolysis related metabolites**			
Glycerol	1.06	0.98 to 1.14	0.14
**Inflammation**			
Glycoprotein acetyls	1.20	1.11 to 1.30	3*10^−6^
**Size of lipoprotein particles (Mean diameters)**			
VLDL particle size	1.12	1.03 to 1.21	0.007
HDL particle size	0.86	0.79 to 0.94	0.0006
**Total lipid concentrations in lipoprotein subclasses**			
Total lipids in chylomicrons and extremely large VLDL	1.09	1.01 to 1.18	0.02
Total lipids in very large VLDL	1.09	1.01 to 1.17	0.03
Total lipids in large VLDL	1.12	1.03 to 1.21	0.005
Total lipids in medium VLDL	1.12	1.04 to 1.21	0.004
Total lipids in IDL	1.07	0.99 to 1.16	0.003
Total lipids in large LDL	1.09	1.01 to 1.18	0.01
Total lipids in medium LDL	1.09	1.01 to 1.18	0.08
Total lipids in small LDL	1.08	1.00 to 1.17	0.03
Total lipids in small HDL	0.97	0.90 to 1.05	0.02
**Cholesterol**			
Cholesterol in VLDL	1.09	1.01 to 1.18	0.03
Remnant cholesterol (non-HDL, non-LDL -cholesterol)	1.07	0.99 to 1.16	0.07
Free cholesterol	1.04	0.96 to 1.12	0.36
**Glycerides and other Phospholipids**			
Serum total TG	1.15	1.07 to 1.24	0.0002
TG in VLDL	1.14	1.05 to 1.23	0.0013
TG in LDL	1.19	1.11 to 1.28	3*10^−6^
TG in HDL	1.13	1.04 to 1.22	0.0021
Sphingomyelins	0.98	0.90 to 1.06	0.56
**Fatty Acids (FA)**			
Saturated FA	1.09	1.01 to 1.18	0.02
Monounsaturated FA	1.12	1.04 to 1.21	0.004
Polyunsaturated FA	1.05	0.97 to 1.13	0.26
Omega-3 FA	0.97	0.90 to 1.05	0.43
docosahexaenoic acid	0.96	0.89 to 1.04	0.33
Omega-6 FA	1.06	0.98 to 1.14	0.14
linoleic acid	1.06	0.98 to 1.14	0.14
Conjugated linoleic acid	1.05	0.97 to 1.13	0.23
**Fatty acids ratios, relative to total fatty acids**			
Estimated degree of unsaturation	0.90	0.83 to 0.97	0.009
Ratio of saturated FA to total FA (%)	1.01	0.93 to 1.08	0.90
Ratio of monounsaturated FA to total FA (%)	1.11	1.03 to 1.21	0.007
Ratio of polyunsaturated FA to total FA (%)	0.90	0.84 to 0.97	0.009
Ratio of omega-3 FA to total FA (%)	0.90	0.83 to 0.97	0.008
Ratio of docosahexaenoic acid to total FA (%)	0.90	0.83 to 0.97	0.009
Ratio of omega-6 FA to total FA (%)	0.93	0.87 to 1.01	0.083
Ratio of linoleic acid to total FA (%)	0.96	0.89 to 1.04	0.28
Ratio of conjugated linoleic acid to total FA (%)	1.03	0.95 to 1.11	0.53

^*^Cox regression models were performed to estimate association between each metabolites and risk of CVD onset over the 16-y of follow-up. Models were adjusted for age, sex, total energy intake, ethnicity, smoking habits, physical activity, type 2 diabetes, diastolic and systolic blood pressure, use of antihypertensive drugs and use of lipid-lowering drugs.

**Figure 3:**
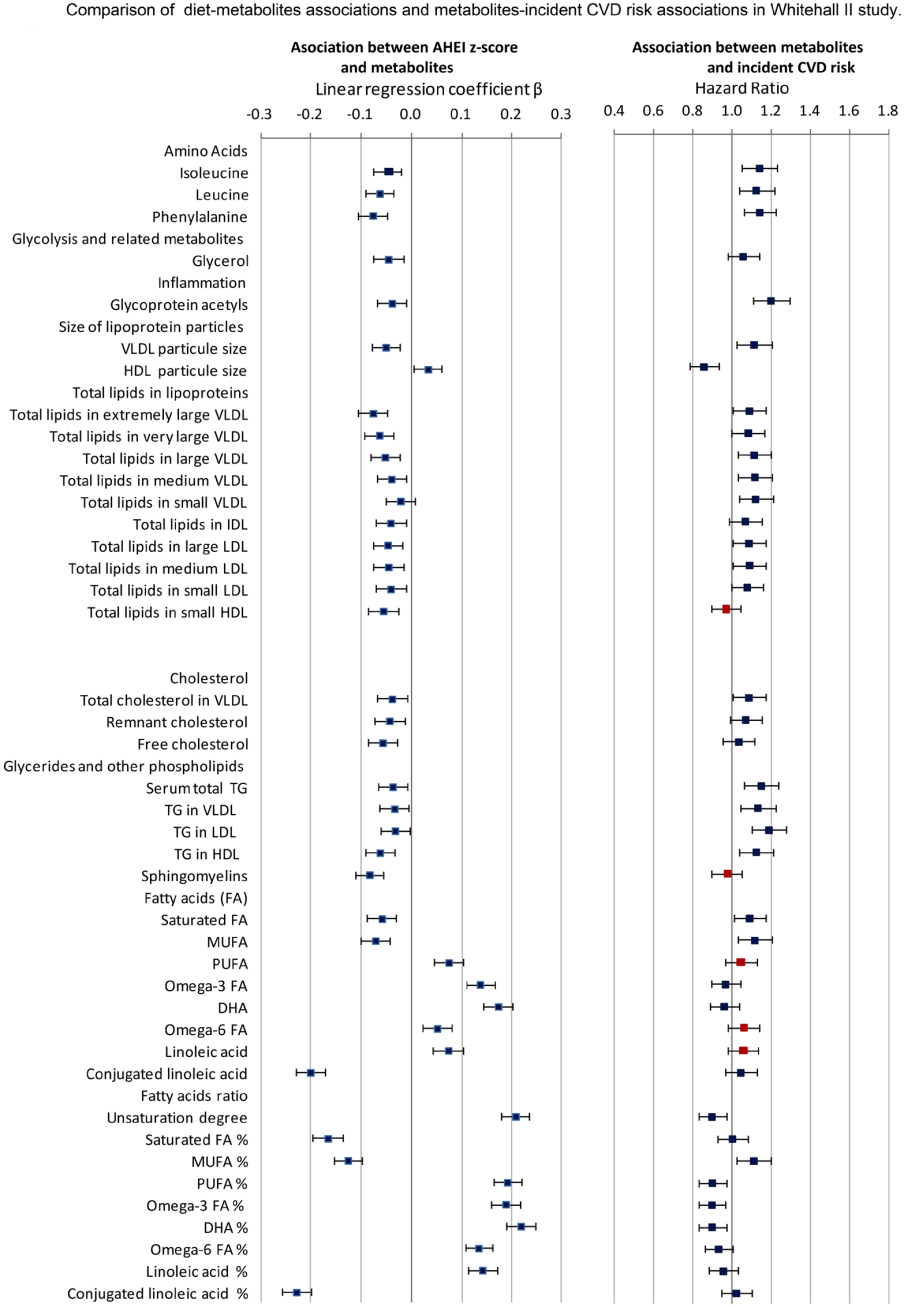
Comparison of diet-metabolites associations and metabolites-incident CVD risk associations in Whitehall II study.  Associations directionally concordant.  Associations directionally discordant. On the left hand size: Linear regression models estimating the associations between AHEI z-score and the 41 selected metabolites performed in 4824 participants and adjusted for age, sex, total energy intake, ethnicity, smoking habits, physical activity, type 2 diabetes, diastolic and systolic blood pressure, use of antihypertensive drugs and use of lipid-lowering drugs. Results are expressed as linear regression coefficients accompanied with their 95% confidence interval. To facilitate comparison, metabolites were first square root transformed and then standardized to z-scores (mean = 0, SD = 1). On the right hand size: Cox proportional hazards regression models estimating the association between the selected 41 metabolites and the risk of incident CVD over the 15.8 years of follow-up, performed in 5840 Whitehall II participant, adjusted for age, sex, total energy intake, ethnicity, smoking habits, physical activity, type 2 diabetes, diastolic and systolic blood pressure, use of antihypertensive medication. Results are expressed as Hazard Ratio accompanied with their 95% confidence interval. To facilitate comparison, metabolites were first square root transformed and then standardized to z-scores (mean = 0, SD = 1).

Figure 3 illustrates whether metabolites associated with poor adherence to healthy dietary guidelines were also related to higher CVD risk. Of the 41 diet-metabolites and metabolites-CVD risk associations assessed, only 5 were directionally discordant and concerned polyunsaturated fatty acids, omega 6 and linoleic acids whose higher blood concentrations were associated with higher CVD risk but without reaching statistical significance. Discordance in terms of direction of association was also observed for total lipids in small HDL and sphingomyelin (Fig. 3).

## Discussion

**Figure 4:**
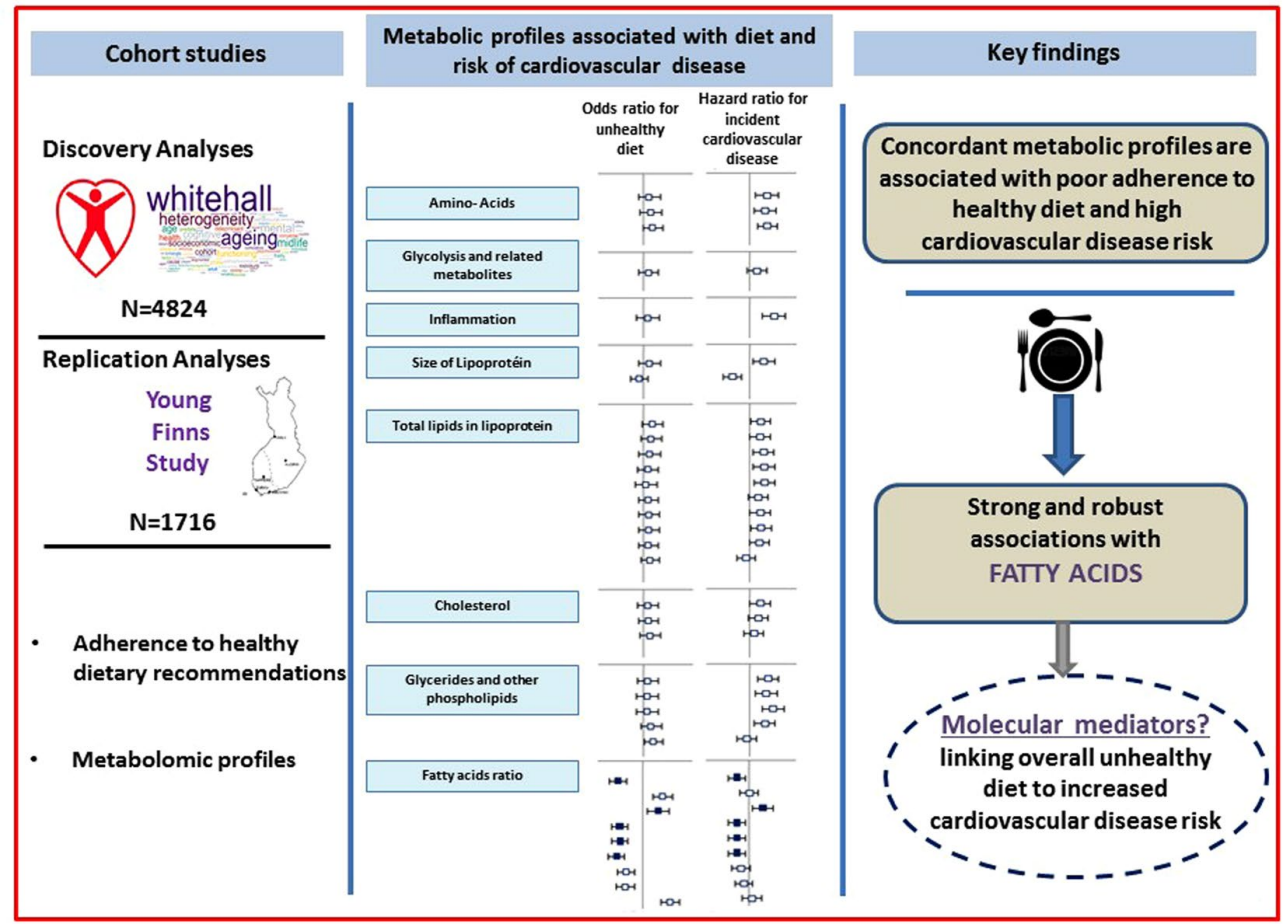
Metabolomic profiles associated with low adherence to healthy dietary guidelines and with the risk of incident cardiovascular diseases -. The metabolic profiling analyses identified 41 metabolites associated with the adherence to healthy diet in Whitehall II study. Replication analyses in the Young Finns Study showed that most of these diet-metabolites associations were directionally concordant. We then assessed the extent to which each of the 41 metabolites associated with diet score also predicted CVD events over the 15.8 years of Whitehall II Study follow-up. Results showed that most of metabolites associated with poor adherence to healthy dietary guidelines are also related to higher CVD risk and consisted of amino acids, glycoprotein acetyls, size of lipoprotein particule size, lipids in lipoproteins, cholesterol and triglycerides and fatty acids. These findings highlight a specific fatty acid patterns robustly associated with both adherence to healthy diet and reduced risk of CVD. These specific fatty acids pattern consisted of lower levels of saturated and monounsaturated fatty acids and higher ratio of polyunsaturated fatty acids, omega-3 and docosahexaenoic acid relative to total fatty acids concentrations, possibly representing a molecular link between healthy diet and lower cardiovascular disease risk.

The present study based on metabolic profiling analyses identified and replicated metabolites associated with the adherence to dietary recommendations provided by the Alternative Healthy Eating Index after taking into account potential confounders and multiple testing in two population-based studies - the Whitehall II and the Cardiovascular Risk in Young Finns. A key finding of these analyses concerns the metabolic profiles of fatty acids associated with diet score. Furthermore, our study highlights the concordance between metabolites profile associated with low adherence to healthy diet and the metabolites profile associated with 15.8-year risk of CVD in Whitehall II participants by showing that an increased risk of CVD onset was associated with high levels of saturated and monounsaturated fatty acids and a decreased risk of CVD was associated with a higher ratio of polyunsaturated fatty acids, omega-3 and docosahexaenoic acid relative to total fatty acids concentrations (Fig. 4).

Our metabolic profiling analyses identified 41 metabolites associated with the adherence to healthy diet. The strongest associations between metabolites and AHEI score were observed for fatty acid measures. We reported a robust and positive association between AHEI scores and degree of unsaturation of fatty acids, ratio and concentrations of polyunsaturated fatty acids including omega-3 (docosahexaenoic acid in particular, brought by fatty fish intake but also oil supplements), omega-6 (linoleic acids found in nuts, fatty seeds and their derived vegetable oil). Conversely, a negative association was found between AHEI scores and ratio (and concentrations) of saturated (found in dairy products, fatty products, processed food and fatty meat intakes) and monounsaturated fatty acids (affected by vegetable oils, lean meat but also produced endogenously by the desaturation of dietary saturated fatty acids^4,14^) and conjugated linoleic acids (found in ruminant meat and dairy products^15^). This fatty acids pattern associated with AHEI score was directionally concordant with fatty acids pattern (except for omega-6) associated with incident CVD in Whitehall II study. Our results are also concordant with previous findings from observational studies suggesting associations of higher levels of omega-3^16^ and linoleic acid^17^ with lower coronary heart disease events and an increased disease risk in relation to high levels of monounsaturated fatty acids^4,18^. The strong association found between fatty acids and diet in the Whitehall II and Young Finns studies and the fact that similar metabolic profile of fatty acids was associated with incident CVD suggest that these specific fatty acids are potential molecular mediators between unhealthy diet and increased CVD risk. Even if recent randomized trials^19,20^ did not indicate a beneficial impact of replacing dietary saturated fatty acids with polyunsaturated ones on CVD risk, our work suggests that the better understanding of the mechanisms underlying the variability of these fatty acids may be helpful in explaining how overall diet might be linked to CVD development.

We identified detailed lipid profiles associated with a good adherence to AHEI recommendations. Using NMR spectroscopy, we were able to determine the lipoprotein subclasses distribution as well as their lipid composition. We found that participants with high score in AHEI had a lipid profile characterized by lower concentrations of lipids in chylomicrons and extremely large, very large and large VLDL, as well as small HDL compared to participants with low score in AHEI. Higher amounts of lipids packaged into chylomicrons may reflect higher ingestion of lipids through the diet and postprandial lipidemia, an established risk factor for CVD^21,22^. Since chylomicrons and VLDL are competitive substrates for triglyceride hydrolysis by lipoprotein lipase in adipose and muscle tissues, higher amounts of circulating chylomicrons are usually associated with predominance of oversized VLDL particles. This specific metabolic profile of lipids in participants with low AHEI scores has also been linked to an increased risk of artherosclerosis and premature CVD^23–25^. The predominance of large VLDL has also been linked to metabolically unhealthy individuals, regardless of BMI and metabolic health definition^21^.

Even if the NMR metabolomics platform featured here is not designed for novel biomarker discovery and includes less metabolites than mass spectrometry-based platforms, the panel of biomarkers covers a wide range of potential relevant biomarkers for diet-CVD associations, including amino acids, glycolysis related metabolites, inflammation, lipids and cholesterol, glycerides and other phospholipids, and fatty acids. The possibility to quantify these measures robustly in a single experiment^26^ is important to determine their relative importance for diet and CVD risk.

In contrast to other NMR methodologies of advanced lipoprotein profiling^27^, the platform used in this study provides quantification of many fatty-acid measures, some abundant proteins, and a broad range of low-molecular-weight metabolites together with very detailed lipoprotein subclasses profiling^28^. This simultaneous quantification of circulating biomarkers across multiple pathways provide a very detailed picture of a person’s metabolic state^27^; we found that in particular fatty acids and lipids components metabolites play a role in both overall unhealthy diet and incidence of CVD events.

Beyond the lipid and fatty acids components, we showed that amino acid components – phenylalanine, leucine and isoleucine - were also associated with both lower AHEI score and increased incident CVD risk. These amino acids have previously been associated with higher risks of developing type 2 diabetes^29–31^. Branched-chain and aromatic amino acid are affected by intakes of animal (pork, beef, chicken, eggs and dairy products) and plant (soy beans, rice, corn, wheat) protein^32^. However, our analyses did not allow to assess the associations of these amino acids in the diet-CVD association according to their plant or animal origins. Further analyses to examine this question would be relevant in a context where beneficial effects of plant protein on cardiometabolic diseases has been reported^33^.

Our study has both strengths and limitations. First, the assessment of dietary intake using a semi-quantitative food frequency questionnaire covered only specific foods and is recognized to be less precise than dietary assessment by the food diary method. However, in a large sample size cohort study, the use of food frequency questionnaires is particularly adapted and a commonly used method. Second, we assessed healthy diet through using the AHEI score which is a summary measure of the degree to which an individual’s diet conforms to the serving recommendations of the US Department of Agriculture Food Guide Pyramid and the US Dietary Guidelines for Americans^11^. By being based on a set of specific and limited food groups, AHEI does not cover all aspects of “healthy” diet and may not be adapted to dietary habits in all populations. However, high scores on this index have been shown to be associated with reduced risk of CVD^11^, and type 2 diabetes^34^. The use of AHEI in the present analyses is particularly relevant, as previous findings from the Whitehall II study suggest that adherence to the AHEI may reduce the long-term risk of all-cause and cardiovascular mortality^12^ and to be related to an almost 2-fold higher odds of reversing the metabolic syndrome^35^, a condition known to predict cardiovascular morbidity and mortality^36^. Third, AHEI provides an overall measure of the extent to which a person adheres healthy dietary guidelines in terms of the intake of vegetables, fruits, nuts and soy, white vs red meat, trans-fat, polyunsaturated and saturated fatty acids, multivitamin, alcohol and cereal fiber. Fourth, to counteract the problem of multiple comparisons we applied a stringent Bonferroni correction which reduces the probability of false significant findings but might increase the probability of false negative results, since many of the examined metabolites and lipid components are strongly correlated with one another. Additionally, we adjusted our analyses for correlated measures such as blood pressure that may artificially reduce the associations’ estimates. Fifth, with an epidemiological observational framework, our observations may be partly explained by unmeasured confounders such as gut microbiota which can potentially influence metabolite variability as well as dietary behaviors. However, by carrying out our analyses on a larger sample size population study compared to previous studies on the same topic and by replicating our findings in another cohort study while previous reports were based on single cohort studies, bring strength to the validity of our observations. The NMR platform used has also limitations. The metabolic profile measured through this platform provided fasting steady-state levels of metabolites. The fact that metabolites related to carbohydrate and protein intakes might be less detectable in fasting state than lipids and fatty acids might explain why metabolites found to be associated with high quality diet were lipids and fatty acids while significant associations with branched amino acids, metabolites related to glycolysis were scarce and most of them were not confirmed in the replication analyses. Furthermore, as glycolysis related metabolites and some amino-acids, lipids and specific fatty acids are produced endogenously with different rates depending on issues, such as individual’s metabolic state, the NMR metabolite measures reflect both metabolites’ exogeneous intake and their endogenous synthesis; they cannot be viewed as markers of specific dietary intakes. A further limitation is that the NMR platform does not include many metabolites from vegetables, fruits, nuts and soy. Further research examining the association of dietary exposure to a wider range of metabolites is needed.

## Conclusions

Our metabolic profiling study enabled us to identify and replicate a number of metabolites robustly associated with adherence to dietary recommendations provided by the Alternative Healthy Eating Index. A key finding of these analyses concerns the metabolic profiles of fatty acids (higher ratio of polyunsaturated fatty acids, omega-3, omega-6 and lower ratio of saturated, monounsaturated and conjugated fatty acids relative to total fatty acids) associated with AHEI score in Whitehall II Study and in Young Finns Study. Our report also highlights the high overlap in metabolites associated with low adherence to healthy dietary guidelines and those predicting long-term risk of CVD in Whitehall II. By showing that an increased risk of CVD onset was associated with high levels of saturated and monounsaturated fatty acids and a decreased risk of CVD was associated with higher ratio of polyunsaturated fatty acids, omega-3 and docosahexaenoic acid relative to total fatty acids concentrations, our findings suggest that these specific fatty acids might be important molecular mediators linking overall unhealthy diet to increased CVD risk.

## Methods

### Study samples

Participants of the discovery cohort were drawn from the Whitehall II cohort study^10^, an on-going prospective cohort study of adults recruited from 20 London-based Civil Service departments in 1985^10^. Of these, 10 308 (6,895 men and 3,413 women, aged 35 to 55) enrolled, a response proportion of 73%. The baseline medical examination (phase 1) took place during 1985/88, and subsequent phases including both clinical examination and self-administrated questionnaire have taken place approximately every 5 years. The subjects included in the metabolites-diet association analyses (n = 4824) was a sample of men (n = 3483) and women (n = 1341) who participated in the 1997/99 clinical examination, and whose serum sample was profiled using NMR metabolomics and had complete data on diet and covariates assessed in 1997/99. Participants gave full informed written consent to participate in the study and ethical approval was obtained from the University College London Hospital committee on the Ethics of Human Research. All research was performed in accordance with relevant guidelines/regulations.

Replication analyses were based on the 2001 survey of the Cardiovascular Risk in Young Finns Study originally designed to study associations of childhood risk factors to disease in adulthood (youngfinnsstudy.utu.fi)^13^. The baseline study conducted in 1980 included n = 3596 children and adolescents aged 3–18. The 2283 individuals participating in 2001 survey (response rate 64%)^13,37^ were representative of the baseline cohort^13^. Among these, n = 2247 individuals provided an overnight fasting blood samples, and the resulting serum samples were stored at −80 °C prior to metabolic profiling by serum NMR metabolomics which was complete for 2161 participants. We further excluded from the present analyses participants with missing data on dietary variables, and main covariates including age sex, total energy intake, alcohol consumption, smoking status, physical activity index assessed by metabolic equivalent of task, systolic and diastolic blood pressure (mm Hg), use of antihypertensive drugs and type 2 diabetes. Assessment of these variables have been described elsewhere^13,38,39^. All participants gave written informed consent, and the study was approved by the ethics committees of each of the five participating medical university sites in Finland.

**Figure 5:**
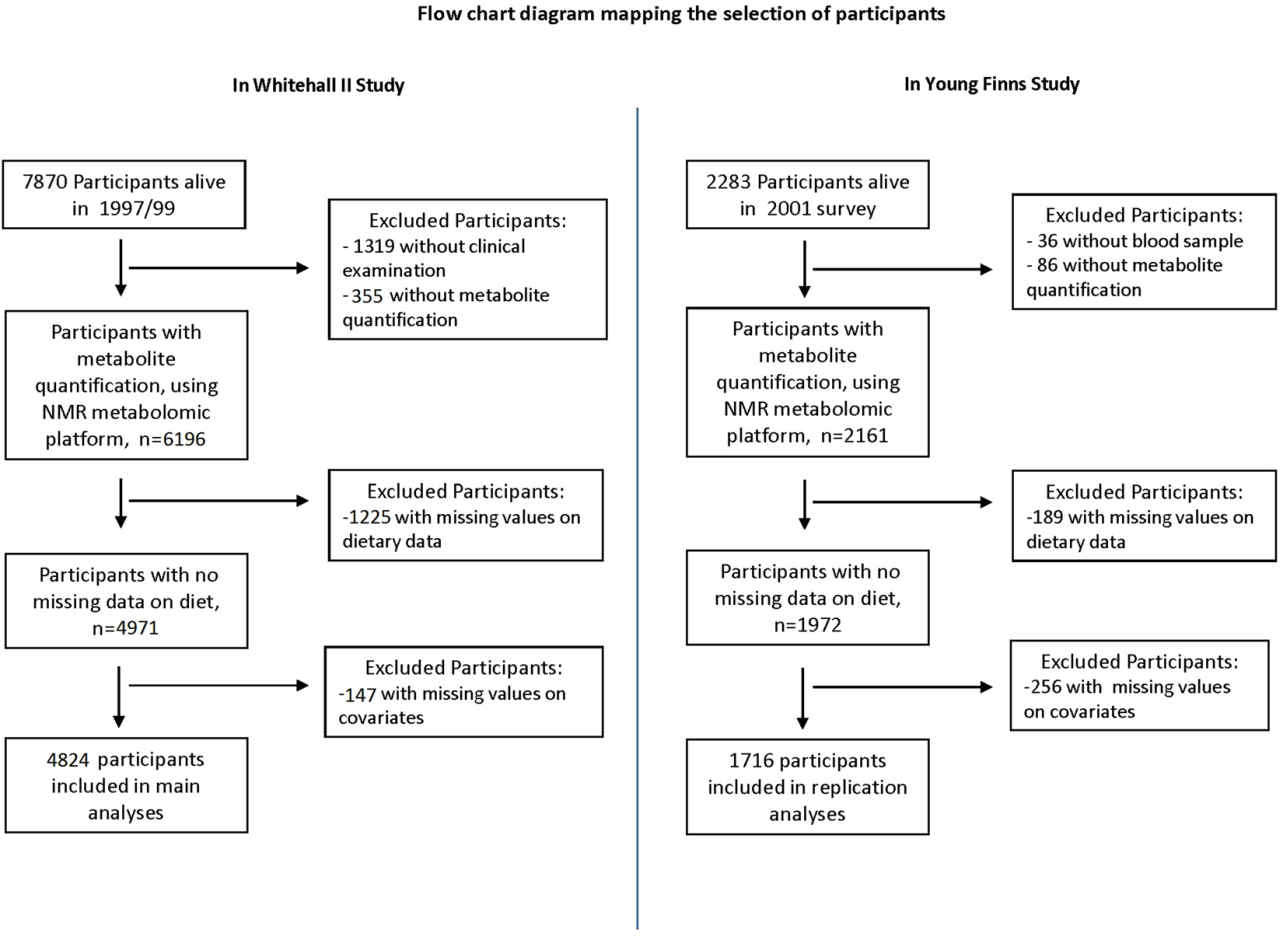
Flow chart diagram mapping the inclusion of Whitehall II and Young Finns Study participants.

The flow chart diagrams mapping the selection of Whitehall II and Young Finns Study participants are provided in Fig. 5.

### Assessment of clinical characteristics

In the Whitehall II study, socio-demographic, health behaviors and health status factors assessed in 1997/99 were considered. Socio-demographic factors included sex, age and ethnicity (white/South Asian/Black). Health behaviors consisted of smoking status (current/former/non smoker), total energy intake (in kcal per day, estimated from the food frequency questionnaire) and physical activity. Based on the physical activity questionnaire that consisted of 20 items on frequency and duration of participation in walking, cycling, sports, gardening, housework, and home maintenance, frequency and duration of each activity were combined to compute Metabolic Equivalent of Task (MET) units/hours/week of moderate to vigorous physical activity^40^. Health status factors considered were those related to cardiovascular risk factors. They included measures of systolic and diastolic blood pressure, use of antihypertensive drugs; type 2 diabetes (diagnosed according to the WHO definition);and use of lipid-lowering drugs. In the Young Finns Study, corresponding assessment of socio-demographic, health behaviors and clinical characteristics was undertaken^13,38^.

### Metabolite quantification

A high-throughput NMR metabolomics platform^28^ was used for the quantification of metabolites from serum samples^4^. We focused on 80 lipid and abundant metabolite measures listed in Supplemental Material-Table A. All metabolites were measured in a single experimental setup that allows for the simultaneous quantification of both routine lipids, total lipid concentrations of 14 lipoprotein subclasses, fatty acid composition such as MUFA and PUFA, various glycolysis precursors, ketone bodies, and amino acids in absolute concentration units. The NMR metabolomics platform has previously been used in various epidemiological studies^4,41^, details of the experimentation have been described^4^ and the method has recently been reviewed^28,42,43^.

### Dietary assessment

Dietary intake was assessed using a semi-quantitative food-frequency questionnaire (FFQ) including 127 food items as described previously^12,44^. The validity and reliability of the FFQ in terms of nutrients and food consumption have been documented in detail elsewhere^44,45^. The AHEI score^11^ - a score reflecting dietary guidelines adapted to the UK framework - was implemented in Whitehall II and Young Finns Study cohorts. It was based on the intake of 9 dietary components: (1) vegetables, (2) fruits, (3) nuts and soy, (4) the ratio of white (seafood and poultry) to red meat, (5) trans-fat, (6) the ratio of polyunsaturated to saturated fatty acids, (7) long-term multivitamin use (<5 or ≥5 y), (8) alcohol consumption and (9) cereal fiber. Each component had the potential to contribute 0 to 10 points to the total score, with the exception of multivitamin use, which contributed either 2.5 or 7.5 points. All the component scores were summed to obtain a total AHEI score ranging from 2.5 to 87.5 with higher scores denoting a healthier diet. Means of AHEI score and its components for both cohorts are detailed in Supplementary Material-Table I. AHEI was defined *a priori* based upon previous knowledge. In 2012, a new measure of the AHEI has been proposed – the AHEI 2010. This index has also been implemented, It includes 11 components, its distribution is detailed in Supplementary Material-Table J.

### Ascertainment of incident cardiovascular disease

Whitehall II participants were linked to electronic medical records to ascertain cardiovascular disease, including coronary heart disease and stroke. Records for the first included hospitalisations from coronary heart disease as a primary or secondary diagnosis (defined using ICD-9 codes 410-414 and ICD-10 codes I20-I25 or procedures K40-K49, K50, K75, U19) and coronary deaths (defined using ICD-9 codes 410-414 and ICD-10 codes I20-I25 in death certificates). Data on stroke included records on hospitalizations due to stroke as a primary or secondary diagnosis and stroke deaths (defined using ICD-9 codes 430, 431, 434, 436 and ICD-10-codes I60, I61, I63, I64). The Young Finns Study participants were too young to have CVD events (less than 20 events during the 12 years follow-up).

### Statistical analyses

All metabolite concentrations were squared root transformed prior to analyses to obtain approximately normal distribution. The metabolite measures were subsequently standardized using z-score (mean = 0, standard deviation = 1). The overall AHEI scores, normally distributed in the two cohorts, were analyzed as continuous variable using z-scores too. Associations between AHEI z-score and each metabolite were assessed by performing linear regression models first adjusted for age, sex and total energy intake. Metabolites found significantly associated with AHEI score at p < 0.0006 (Bonferroni correction of p < 0.05 accounting for 80 independent tests) were selected for further testing, including replication analysis and associations with CVD event risk. For the selected metabolites, linear regression models further adjusted for ethnicity, smoking habits, physical activity, systolic and diastolic blood pressure, use of antihypertensive drugs, type 2 diabetes and use of lipid-lowering drugs were performed. These analyses were repeated after taking into account BMI. In sensitivity analyses these multivariable adjusted models estimating the association between AHEI z-score and metabolites were repeated (1) in participants free of cardiovascular diseases in 1997/99 (i.e. clinically verified non-fatal myocardial infarction or definite angina), (2) in participants without prevalent cancer and (3) after excluding participants who self-reported longstanding illness.

To examine whether the findings of the associations between AHEI and metabolites in Whitehall II study were replicable, we used data from the Young Finns Study and applied similar multivariable linear regression models. As for analyses in Whitehall II, metabolites were square rooted and z-scores were computed and AHEI was treated as a z-score. The results from individual cohorts were then combined by using inverse variance fixed effect meta-analysis.

To assess the extent to which metabolites associated with diet score were also those predictive of CVD events, we conducted Cox proportional hazards regression models for each of the selected metabolites as predictors of incident CVD events (adjusted for similar risk factors as those considered in the above-mentioned analyses). To do so we selected the 5840 Whitehall II participants for whom quantification of metabolites and clinical characteristics were available in 1997/99 and a follow-up of cardiovascular diseases over the 16-year follow-up. Participants with prevalent CVD were excluded to concentrate on associations with first onset of CVD.

### Data availability

The datasets generated during and/or analysed during the current study are available from the corresponding author on reasonable request.

## Electronic supplementary material

Supplementary Material
