# Supplementary material for: Association of circulating metabolites with healthy diet and risk of cardiovascular disease: analysis of two cohort studies
Source: Sci Rep. 2018 Jun 5;8:8620. doi: 10.1038/s41598-018-26441-1 (PMC5988716; doi:10.1038/s41598-018-26441-1)
Supplement: Supplementary file 1 — Supplementary Material [file 41598_2018_26441_MOESM1_ESM.pdf]

Manuscript Title : Association of circulating metabolites with healthy diet and risk of cardiovascular disease: analysis of two cohort studies

Authors : Tasnime N. Akbaraly, Peter Würtz, Archana Singh-Manoux, Martin J. Shipley, Rita Haapakoski, Maili Lehto, Catherine Desrumaux, Mika Kähönen, Terho Lehtimäki, Vera Mikkilä, Aroon Hingorani, Steve E. Humphries, Antti J. Kangas, Pasi Soininen, Olli Raitakari, Mika Ala-Korpela, Mika Kivimäki

Online Supplementary Material

Supplementary Table A: Metabolite concentrations in Whitehall II participants

	N	Mean	sd
**Amino Acids**			
Alanine (mmol/L)	4797	0.42	0.06
Glutamine (mmol/L)	4784	0.60	0.07
Glycine (mmol/L)	4787	0.30	0.05
Histidine (mmol/L)	4734	0.08	0.01
Isoleucine (mmol/L)	4821	0.06	0.01
Leucine (mmol/L)	4819	0.09	0.02
Valine (mmol/L)	4809	0.19	0.03
Phenylalanine (mmol/L)	4800	0.08	0.01
Tyrosine (mmol/L)	4776	0.06	0.01
**Glycolysis related metabolites**			
Glucose (mmol/L)	4745	5.17	1.17
Lactate (mmol/L)	4816	1.91	0.55
Pyruvate (mmol/L)	4815	0.09	0.03
Citrate (mmol/L)	4798	0.13	0.02
Glycerol (mmol/L)	4767	0.10	0.04
**Ketone bodies**		4745	
Acetate (mmol/L)	4818	0.07	0.03
Acetoacetate (mmol/L)	4820	0.06	0.04
3-hydroxybutyrate (mmol/L)	4806	0.16	0.11
**Fluid balance**			
Creatinine (mmol/L)	4758	0.08	0.01
Albumin (standardized concentration units)	4817	0.10	0.01

**Inflammation**			
Glycoprotein acetyls, mainly a1-acid glycoprotein (mmol/L)	4821	1.44	0.22
VLDL particles (nm)	4824	35.9	1.0
LDL particles (nm)	4824	23.6	0.1
HDL particles (nm)	4824	9.9	0.2
Total lipids in chylomicrons and extremely large VLDL (mmol/L)	4805	0.024	0.016
Total lipids in very large VLDL (mmol/L)	4804	0.046	0.039
Total lipids in large VLDL (mmol/L)	4809	0.186	0.136
Total lipids in medium VLDL (mmol/L)	4811	0.499	0.248
Total lipids in small VLDL (mmol/L)	4807	0.666	0.216
Total lipids in very small VLDL (mmol/L)	4808	0.642	0.145
Total lipids in IDL (mmol/L)	4815	1.320	0.277
Total lipids in large LDL (mmol/L)	4816	1.479	0.336
Total lipids in medium LDL (mmol/L)	4812	0.835	0.203
Total lipids in small LDL (mmol/L)	4815	0.528	0.123
Total lipids in very large HDL (mmol/L)	4809	0.459	0.196
Total lipids in large HDL (mmol/L)	4809	0.755	0.297
Total lipids in medium HDL (mmol/L)	4817	0.961	0.185
Total lipids in small HDL (mmol/L)	4808	1.165	0.136
**Cholesterol**			
Serum total cholesterol (mmol/L)	4812	5.07	0.92
Total cholesterol in VLDL (mmol/L)	4797	0.82	0.25
Total cholesterol in IDL (mmol/L)	4808	0.84	0.19
Total cholesterol in LDL (mmol/L)	4812	1.89	0.49
Total cholesterol in HDL (mmol/L)	4809	1.51	0.32
Total cholesterol in HDL2 (mmol/L)	4824	0.99	0.29
Total cholesterol in HDL3 (mmol/L)	4809	0.52	0.07
Remnant cholesterol (non-HDL, non-LDL -cholesterol) (mmol/L)	4806	1.66	0.39
Esterified cholesterol (mmol/L)	4743	3.65	0.68
Free cholesterol (mmol/L)	4743	1.41	0.26
**Glycerides and other Phospholipids**			
Serum total TG (mmol/L)	4806	1.24	0.47
TGin VLDL (mmol/L)	4808	0.76	0.37
TG in LDL (mmol/L)	4805	0.21	0.06
TG in HDL (mmol/L)	4805	0.14	0.04
Diacylglycerol (mmol/L)	4678	0.02	0.01
Ratio of diacylglycerol to TG	4678	0.02	0.01
Total phosphoglycerides (mmol/L)	4742	2.02	0.38

Ratio of TG to phosphoglycerides	4742	0.57	0.23
Phosphatidylcholine and other cholines (mmol/L)	4741	1.99	0.37
Sphingomyelins (mmol/L)	4743	0.49	0.09
Total cholines (mmol/L)	4737	2.39	0.40
**Apolipoproteins (APO)**			
APO A-I (g/L)	4824	1.60	0.19
APO B (g/L)	3687	101.96	25.18
Ratio of APOB to APOA-I	4824	0.61	0.13
**Fatty Acids (FA)**			
Total FA (mmol/L)	4742	12.25	2.44
Estimated description of fatty acid chain length, not actual carbon number	4740	17.19	0.31
Saturated FA (mmol/L)	4737	4.65	0.96
Monounsaturated FA; 16:1, 18:1 (mmol/L)	4737	2.97	0.87
Polyunsaturated FA (mmol/L)	4742	4.63	0.81
Omega-3 FA (mmol/L)	4743	0.53	0.16
22:6, docosahexaenoic acid (mmol/L)	4743	0.19	0.07
Omega-6 FA (mmol/L)	4743	4.10	0.73
18:2, linoleic acid (mmol/L)	4743	3.33	0.65
Conjugated linoleic acid (mmol/L)	4741	0.05	0.03
**Fatty acids ratios relative to total fatty acids**			
Estimated degree of unsaturation	4741	1.21	0.07
Ratio of saturated FA to total FA (%)	4736	37.94	1.72
Ratio of monounsaturated FA to total FA (%)	4736	24.00	3.07
Ratio of polyunsaturated FA to total FA (%)	4741	38.07	3.36
Ratio of omega-3 FA to total FA (%)	4742	4.34	1.10
Ratio of 22:6 docosahexaenoic acid to total FA (%)	4742	1.58	0.47
Ratio of omega-6 FA to total FA (%)	4742	33.73	3.25
Ratio of 18:2 linoleic acid to total FA (%)	4742	27.44	3.48
Ratio of conjugated linoleic acid to total FA (%)	4740	0.39	0.17

Supplementary Table B:Comparison of characteristics assessed in 1997/99 between Whitehall II participants included in the analyses and those excluded.

		Participants included, n=4824	Participants excluded, n=3046	
**Characteristics of participants in 1997/99**		**N (%) or (mean ±SD)***	**N (%) or (mean ±SD)***	**P-value†**
Sex	Men	3483 (72.2)	1990 (65.6)	<0.001
	Women	1341 (27.8)	1056 (34.4)	
Age, years		4824 (55.9 ±6.1)*	3046 (56.0 ±6.0)*	<0.001
Ethnicity	White	4541(93.9)	2645 (89.1)	<0.001
Smoking habits	Current	470 (8.9)	500 (14.6)	<0.001
Physical activity, hours		4824 (3.49 ±3.23)*	2290 (2.91 ± 3.02)*	<0.001
Total Energy intake, kcal/day)		4824 (2233±683)*	593 (2114 ±771)*	<0.001
**CVD Risk factors**				
Type 2 diabetes	Yes	296 (6.1)	205 (6.1)	0.22
Systolic Blood pressure, mmHg		4824 (123.1 ±16.1)*	1515 (123.4 ±16.6)*	0.48
Diastolic Blood pressure, mmHg		4824 (77.4±10.5)*	1715 (78.3±10.7)*	0.003
Use of antihypertensive drugs	Yes	607 (12.6)	491 (16.4)	<0.001
Total Cholesterol, mmol/L		4824 (5.93 ±1.04)*	1644 (5.94 ± 1.10)*	0.60
Triglycerides, mmol/L		4823 (1.35 ± 0.86)*	1644 (1.46 ± 0.98)*	<0.001
HDL-Cholesterol, mmol/L		4298 (1.46 ±0.39)*	1448 (1.45 ±0.40)*	0.54
Use of lipids lowering drugs	Yes	155 (3.1)	116 (4.0)	0.05
**AHEI score**				
Low AHEI score (<51.5 points)		2385(49.4)	200 (47.5)	0.39
High AHEI score (≥51.5 points)		2439 (50.6)	221 (52.5)	

Supplementary Table C:Results of linear regression models estimating the association between AHEI z-score and each metabolite after adjustment for sex, age and total energy intake in Whitehall II study.

	Beta	Lower range of 95 % CI	Higher range of 95 % CI	p
**Amino Acids**				
Alanine	-0.023	-0.053	0.007	0.13
Glutamine	0.007	-0.022	0.035	0.65
Glycine	0.028	-0.001	0.057	0.06
Histidine	0.002	-0.027	0.031	0.87
Isoleucine	-0.059	-0.086	-0.031	$3*10^{-5}$
Leucine	-0.074	-0.101	-0.047	$1*10^{-7}$
Valine	-0.022	-0.049	0.005	0.12
Phenylalanine	-0.088	-0.117	-0.059	$3*10^{-9}$
Tyrosine	-0.031	-0.060	-0.001	0.04
**Glycolysis related metabolites**				
Glucose	-0.006	-0.036	0.023	0.68
Lactate	-0.040	-0.070	-0.011	0.007
Pyruvate	-0.041	-0.070	-0.011	0.008
Citrate	-0.022	-0.051	0.007	0.14
Glycerol	-0.052	-0.081	-0.022	0.001
**Ketone bodies**				
Acetate	0.005	-0.021	0.030	0.72
Acetoacetate	0.010	-0.019	0.039	0.52
3-hydroxybutyrate	-0.004	-0.033	0.026	0.81
**Fluid balance**				
Creatinine	-0.034	-0.058	-0.010	0.005
Albumin (standardized concentration units)	-0.017	-0.045	0.012	0.26
**Inflammation**				
Glycoprotein acetyls, mainly a1-acid glycoprotein	-0.070	-0.099	-0.041	$3*10^{-6}$
VLDL particles	-0.081	-0.109	-0.053	$1*10^{-8}$
LDL particles	0.014	-0.015	0.044	0.33
HDL particles	0.047	0.021	0.074	0.0005
Total lipids in chylomicrons and extremely large VLDL	-0.104	-0.132	-0.075	$2*10^{-12}$
Total lipids in very large VLDL	-0.094	-0.123	-0.066	$1*10^{-10}$

	Total lipids in large VLDL	-0.085	-0.113	-0.056	$6*10^{-9}$
	Total lipids in medium VLDL	-0.070	-0.099	-0.042	10^{-6}
	Total lipids in small VLDL	-0.051	-0.079	-0.022	0.001
	Total lipids in very small VLDL	-0.046	-0.075	-0.017	0.002
	Total lipids in IDL	-0.052	-0.081	-0.023	0.0004
	Total lipids in large LDL	-0.058	-0.087	-0.029	$8*10^{-5}$
	Total lipids in medium LDL	-0.056	-0.085	-0.027	0.0002
	Total lipids in small LDL	-0.052	-0.082	-0.023	0.0004
	Total lipids in very large HDL	0.005	-0.022	0.032	0.72
	Total lipids in large HDL	0.035	0.008	0.062	0.01
	Total lipids in medium HDL	-0.027	-0.056	0.001	0.06
	Total lipids in small HDL	-0.080	-0.109	-0.050	$1*10^{-7}$
**Cholesterol concentrations**					
	Serum total cholesterol	-0.051	-0.080	-0.021	0.0007
	Total cholesterol in VLDL	-0.062	-0.091	-0.033	$3*10^{-5}$
	Total cholesterol in IDL	-0.046	-0.075	-0.017	0.002
	Total cholesterol in LDL	-0.048	-0.078	-0.019	0.001
	Total cholesterol in HDL	0.006	-0.022	0.033	0.69
	Total cholesterol in HDL2	0.016	-0.012	0.043	0.26
	Total cholesterol in HDL3	-0.032	-0.061	-0.002	0.04
	Remnant cholesterol (non-HDL, non-LDL -cholesterol)	-0.061	-0.090	-0.032	$4*10^{-5}$
	Esterified cholesterol	-0.041	-0.070	-0.012	0.006
	Free cholesterol	-0.072	-0.101	-0.044	$8*10^{-7}$
**Triglyceride (TG) concentrations**					
	Serum total TG	-0.067	-0.097	-0.038	$6*10^{-6}$
	TGin VLDL	-0.063	-0.092	-0.034	$2*10^{-5}$
	TG in LDL	-0.054	-0.083	-0.025	0.0003
	TG in HDL	-0.088	-0.118	-0.059	$4*10^{-9}$
	Diacylglycerol	-0.027	-0.057	0.002	0.07
	Ratio of diacylglycerol to TG	-0.002	-0.032	0.027	0.87
	Total phosphoglycerides	-0.035	-0.064	-0.006	0.02
	Ratio of TG to phosphoglycerides	-0.038	-0.067	-0.009	0.01
	Phosphatidylcholine and other cholines	-0.032	-0.061	-0.004	0.03
	Sphingomyelins	-0.098	-0.126	-0.070	$5*10^{-12}$
	Total cholines	-0.038	-0.066	-0.009	0.009
**Apolipoproteins (APO)**					
	APO A-I	0.001	-0.027	0.029	0.94
	APO B	-0.031	-0.060	-0.002	0.04

Ratio of APOB to APOA-I	-0.032	-0.060	-0.004	0.03
**Fatty Acids (FA)**				
Total FA	-0.048	-0.077	-0.018	0.001
Estimated description of fatty acid chain length, not actual carbon number	-0.003	-0.033	0.026	0.82
Saturated FA	-0.081	-0.111	-0.052	$6*10^{-8}$
Monounsaturated FA; 16:1, 18:1	-0.117	-0.146	-0.087	$7*10^{-15}$
Polyunsaturated FA	0.074	0.045	0.103	$6*10^{-7}$
Omega-3 FA	0.136	0.107	0.165	$2*10^{-20}$
22:6, docosahexaenoic acid	0.175	0.147	0.203	$7*10^{-34}$
Omega-6 FA	0.051	0.022	0.081	0.0006
18:2, linoleic acid	0.074	0.045	0.103	$9*10^{-7}$
Conjugated linoleic acid	-0.230	-0.259	-0.201	$5*10^{-54}$
**Fatty acids ratios relative to total fatty acids**				
Estimated degree of unsaturation	0.258	0.230	0.287	$7 *10^{-70}$
Ratio of saturated FA to total FA	-0.158	-0.187	-0.129	$3*10^{-26}$
Ratio of monounsaturated FA to total FA	-0.186	-0.215	-0.156	$2*10^{-35}$
Ratio of polyunsaturated FA to total FA	0.247	0.219	0.276	$1*10^{-62}$
Ratio of omega-3 FA to total FA	0.210	0.181	0.238	$4*10^{-46}$
Ratio of 22:6 docosahexaenoic acid to total FA	0.240	0.212	0.268	$4*10^{-62}$
Ratio of omega-6 FA to total FA	0.185	0.156	0.214	$2*10^{-35}$
Ratio of 18:2 linoleic acid to total FA	0.181	0.152	0.210	$6*10^{-34}$
Ratio of conjugated linoleic acid to total FA	-0.254	-0.282	-0.225	$1*10^{-65}$

Supplementary Table D: Results of linear regression models estimating the association between AHEI 2010 z-score and each metabolite after adjustment for sex, age and total energy intake in Whitehall II study.

	Beta	Lower range of 95 % CI	Higher range of 95 % CI	p
**Amino Acids**				
Alanine	-0.049	-0.077	-0.020	0.0008
Glutamine	0.030	0.002	0.058	0.03
Glycine	0.028	0.000	0.056	0.05
Histidine	-0.008	-0.036	0.019	0.55
Isoleucine	-0.099	-0.125	-0.072	$3*10^{-13}$
Leucine	-0.106	-0.133	-0.080	$2*10^{-15}$
Valine	-0.043	-0.069	-0.017	0.013
Phenylalanine	-0.092	-0.120	-0.064	$1*10^{-10}$
Tyrosine	-0.058	-0.086	-0.030	$5*10^{-5}$
**Glycolysis related metabolites**				
Glucose	-0.019	-0.047	0.009	0.18
Lactate	-0.053	-0.082	-0.025	0.0002
Pyruvate	-0.079	-0.108	-0.050	$7*10^{-8}$
Citrate	-0.008	-0.036	0.020	0.59
Glycerol	-0.101	-0.129	-0.073	$1*10^{-12}$
**Ketone bodies**				
Acetate	0.006	-0.018	0.031	0.61
Acetoacetate	0.024	-0.004	0.052	0.10
3-hydroxybutyrate	0.002	-0.026	0.030	0.87
**Fluid balance**				
Creatinine	-0.040	-0.063	-0.017	0.0006
Albumin (standardized concentration units)	-0.031	-0.059	-0.003	0.027
**Inflammation**				
Glycoprotein acetyls, mainly a1-acid glycoprotein	-0.119	-0.147	-0.091	$1*10^{-16}$
VLDL particles	-0.121	-0.148	-0.094	$2*10^{-18}$
LDL particles	0.043	0.015	0.071	0.002
HDL particles	0.090	0.065	0.116	$6*10^{-12}$

	Total lipids in chylomicrons and extremely large VLDL	-0.141	-0.168	-0.113	$2*10^{-23}$
	Total lipids in very large VLDL	-0.135	-0.163	-0.108	$8*10^{-22}$
	Total lipids in large VLDL	-0.130	-0.157	-0.102	$2*10^{-20}$
	Total lipids in medium VLDL	-0.117	-0.145	-0.090	$5*10^{-17}$
	Total lipids in small VLDL	-0.109	-0.136	-0.081	$1*10^{-14}$
	Total lipids in very small VLDL	-0.090	-0.118	-0.062	$3*10^{-10}$
	Total lipids in IDL	-0.068	-0.096	-0.040	$1.9*10^{-6}$
	Total lipids in large LDL	-0.080	-0.108	-0.052	$2.4*10^{-8}$
	Total lipids in medium LDL	-0.077	-0.105	-0.049	$6.7*10^{-8}$
	Total lipids in small LDL	-0.074	-0.102	-0.046	$2.6*10^{-7}$
	Total lipids in very large HDL	0.036	0.010	0.062	0.006
	Total lipids in large HDL	0.062	0.036	0.088	$3*10^{-6}$
	Total lipids in medium HDL	-0.061	-0.088	-0.033	$1*10^{-5}$
	Total lipids in small HDL	-0.146	-0.174	-0.118	$4*10^{-24}$
**Cholesterol concentrations**					
	Serum total cholesterol	-0.068	-0.096	-0.040	$1.8*10^{-6}$
	Total cholesterol in VLDL	-0.111	-0.139	-0.083	$9*10^{-15}$
	Total cholesterol in IDL	-0.053	-0.081	-0.025	0.0002
	Total cholesterol in LDL	-0.065	-0.093	-0.037	$6*10^{-6}$
	Total cholesterol in HDL	0.016	-0.010	0.043	0.2
	Total cholesterol in HDL2	0.027	0.000	0.053	0.05
	Total cholesterol in HDL3	-0.026	-0.054	0.003	0.07
	Remnant cholesterol (non-HDL, non-LDL -cholesterol)	-0.094	-0.122	-0.066	$5*10^{-11}$
	Esterified cholesterol	-0.066	-0.094	-0.038	$5*10^{-6}$
	Free cholesterol	-0.071	-0.098	-0.043	$6*10^{-7}$
**Triglyceride (TG) concentrations**					
	Serum total TG	-0.120	-0.148	-0.092	$5*10^{-17}$
	TG in VLDL	-0.116	-0.144	-0.088	$3*10^{-16}$
	TG in LDL	-0.091	-0.119	-0.063	$2*10^{-10}$
	TG in HDL	-0.125	-0.153	-0.096	$6*10^{-18}$
	Diacylglycerol	-0.071	-0.099	-0.042	10^{-6}
	Ratio of diacylglycerol to TG	-0.017	-0.045	0.011	0.24
	Total phosphoglycerides	-0.075	-0.103	-0.047	$1*10^{-7}$
	Ratio of TG to phosphoglycerides	-0.089	-0.117	-0.061	$4*10^{-10}$
	Phosphatidylcholine and other cholines	-0.072	-0.099	-0.044	$4*10^{-7}$
	Sphingomyelins	-0.089	-0.116	-0.062	$1*10^{-10}$
	Total cholines	-0.068	-0.095	-0.040	$1*10^{-6}$
**Apolipoproteins (APO)**					

	APO A-I	0.007	-0.020	0.034	0.61
	APO B	-0.046	-0.074	-0.018	0.001
	Ratio of APOB to APOA-I	-0.051	-0.078	-0.024	0.0002
**Fatty Acids (FA)**					
	Total FA	-0.100	-0.128	-0.072	$4*10^{-12}$
	Estimated description of fatty acid chain length, not actual carbon number	-0.061	-0.090	-0.033	$2*10^{-5}$
	Saturated FA	-0.120	-0.148	-0.092	$1*10^{-16}$
	Monounsaturated FA; 16:1, 18:1	-0.179	-0.207	-0.151	$2*10^{-35}$
	Polyunsaturated FA	0.029	0.001	0.057	0.04
	Omega-3 FA	0.031	0.003	0.059	0.02
	22:6, docosahexaenoic acid	0.067	0.039	0.094	$2*10^{-6}$
	Omega-6 FA	0.024	-0.004	0.053	0.09
	18:2, linoleic acid	0.065	0.037	0.093	$6*10^{-6}$
	Conjugated linoleic acid	-0.221	-0.248	-0.193	10^{-53}
**Fatty acids ratios relative to total fatty acids**					
	Estimated degree of unsaturation	0.209	0.182	0.237	$5*10^{-49}$
	Ratio of saturated FA to total FA	-0.103	-0.131	-0.075	$7*10^{-13}$
	Ratio of monounsaturated FA to total FA	-0.242	-0.269	-0.214	$2*10^{-64}$
	Ratio of polyunsaturated FA to total FA	0.271	0.243	0.298	$9*10^{-81}$
	Ratio of omega-3 FA to total FA	0.122	0.094	0.150	$2*10^{-17}$
	Ratio of 22:6 docosahexaenoic acid to total FA	0.149	0.121	0.176	$4*10^{-26}$
	Ratio of omega-6 FA to total FA	0.238	0.211	0.266	$1*10^{-62}$
	Ratio of 18:2 linoleic acid to total FA	0.246	0.218	0.273	$3*10^{-66}$
	Ratio of conjugated linoleic acid to total FA	-0.221	-0.249	-0.194	$3*10^{-54}$

Supplementary Table E: Associations between AHEI z-score and metabolites before and after adjustment for BMI*

	Before adjustment for BMI		After adjustment for BMI	
	**Beta***	**95% CI**	**Beta***	**95% CI**
**Amino Acids**				
Isoleucine	-0.024	-0.052 to 0.004	-0.025	-0.052 to 0.003
Leucine	-0.043	-0.070 to -0.015	-0.042	-0.070 to -0.015
Phenylalanine	-0.061	-0.091 to -0.030	-0.061	-0.092 to -0.031
**Glycolysis related metabolites**				
Glycerol	-0.036	-0.067 to -0.005	-0.035	-0.066 to -0.004
**Inflammation**				
Glycoprotein acetyls	-0.021	-0.051 to 0.008	-0.023	-0.052 to 0.006
**Size of lipoprotein particles (Mean diameters)**				
VLDL particle size	-0.028	-0.057 to 0.001	-0.030	-0.059 to -0.001
HDL particle size	0.018	-0.010 to 0.047	0.020	-0.009 to 0.048
**Total lipid concentrations in lipoprotein subclasses**				
Total lipids in chylomicrons and extremely large VLDL	-0.055	-0.085 to -0.025	-0.056	-0.086 to -0.026
Total lipids in very large VLDL	-0.043	-0.073 to -0.013	-0.044	-0.074 to -0.014
Total lipids in large VLDL	-0.029	-0.059 to 0.001	-0.030	-0.060 to -0.001
Total lipids in medium VLDL	-0.017	-0.046 to 0.013	-0.018	-0.048 to 0.012
Total lipids in small VLDL	0.001	-0.030 to 0.030	-0.001	-0.031 to 0.029
Total lipids in IDL	-0.031	-0.063 to 0.001	-0.031	-0.063 to 0.001
Total lipids in large LDL	-0.035	-0.068 to -0.003	-0.036	-0.068 to -0.003
Total lipids in medium LDL	-0.033	-0.066 to -0.001	-0.034	-0.066 to -0.001
Total lipids in small LDL	-0.028	-0.061 to 0.004	-0.029	-0.061 to 0.004
Total lipids in small HDL	-0.046	-0.077 to -0.014	-0.045	-0.077 to -0.014
**Cholesterol**				
Cholesterol in VLDL	-0.019	-0.050 to 0.012	-0.020	-0.050 to 0.011
Remnant cholesterol (non-HDL, non-LDL -cholesterol)	-0.028	-0.059 to 0.004	-0.028	-0.060 to 0.004
Free cholesterol	-0.051	-0.083 to -0.019	-0.051	-0.083 to -0.019
**Glycerides and other Phospholipids**				
Serum total TG	-0.015	-0.045 to 0.015	-0.015	-0.045 to 0.015
TG in VLDL	-0.010	-0.040 to 0.019	-0.012	-0.041 to 0.018
TG in LDL	-0.017	-0.048 to 0.014	-0.017	-0.048 to 0.014
TG in HDL	-0.048	-0.079 to -0.017	-0.048	-0.079 to -0.017
Sphingomyelins	-0.078	-0.109 to -0.047	-0.078	-0.109 to -0.047
**Fatty Acids (FA)**				
Saturated FA	-0.044	-0.075 to -0.013	-0.044	-0.075 to -0.013
Monounsaturated FA	-0.054	-0.084 to -0.023	-0.054	-0.084 to -0.023
Polyunsaturated FA	0.083	0.051 to 0.115	0.082	0.050 to 0.114

Omega-3 FA	0.144	0.112 to 0.176	0.144	0.113 to 0.176
docosahexaenoic acid	0.179	0.148 to 0.210	0.179	0.148 to 0.210
Omega-6 FA	0.060	0.028 to 0.092	0.059	0.027 to 0.092
linoleic acid	0.080	0.048 to 0.112	0.079	0.047 to 0.111
Conjugated linoleic acid	-0.182	-0.213 to -0.152	-0.182	-0.213 to -0.152
**Fatty acids ratios, relative to total fatty acids**				
Estimated degree of unsaturation	0.200	0.170 to 0.229	0.200	0.171 to 0.230
Ratio of saturated FA to total FA	-0.162	-0.194 to -0.130	-0.161	-0.194 to -0.129
Ratio of monounsaturated FA to total FA	-0.109	-0.139 to -0.080	-0.109	-0.139 to -0.080
Ratio of polyunsaturated FA to total FA	0.180	0.150 to 0.209	0.179	0.150 to 0.208
Ratio of omega-3 FA to total FA	0.185	0.154 to 0.216	0.186	0.155 to 0.217
Ratio of docosahexaenoic acid to total FA	0.215	0.185 to 0.246	0.216	0.186 to 0.246
Ratio of omega-6 FA to total FA	0.124	0.094 to 0.154	0.124	0.094 to 0.154
Ratio of linoleic acid to total FA	0.132	0.102 to 0.163	0.132	0.102 to 0.162
Ratio of conjugated linoleic acid to total FA	-0.211	-0.242 to -0.180	-0.211	-0.242 to -0.180

Models adjusted for age, sex, total energy intake, ethnicity, smoking habits, physical activity, type 2 diabetes, diastolic and systolic blood pressure, use of antihypertensive drugs and use of lipid-lowering drugs. Results were expressed as linear regression coefficients accompanied with their 95 % confidence interval

* Analyses were carried out on 4175 participants with available data on BMI

Supplementary Table F: Sensitivity analyses of associations between AHEI z-score* and metabolites.

	In Participants without antecedent of CVD N=3920		In Participants without prevalent cancer N=4052	
	Beta	95% CI	Beta	95% CI
**Amino Acids**				
Isoleucine	-0.048	-0.076 to -0.020	-0.043	-0.071 to -0.015
Leucine	-0.064	-0.092 to -0.036	-0.057	-0.084 to -0.029
Phenylalanine	-0.078	-0.109 to -0.048	-0.068	-0.098 to -0.038
**Glycolysis related metabolites**				
Glycerol	-0.043	-0.074 to -0.012	-0.039	-0.069 to -0.009
**Inflammation**				
Glycoprotein acetyls	-0.039	-0.068 to -0.009	-0.036	-0.065 to -0.007
**Size of lipoprotein particles (Mean diameters)**				
VLDL particle size	-0.051	-0.079 to -0.022	-0.053	-0.081 to -0.024
HDL particle size	0.037	0.009 to 0.066	0.034	0.006 to 0.062
**Total lipid concentrations in lipoprotein subclasses**				
Total lipids in chylomicrons and extremely large VLDL	-0.076	-0.105 to -0.046	-0.077	-0.106 to -0.047
Total lipids in very large VLDL	-0.063	-0.093 to -0.034	-0.065	-0.094 to -0.035
Total lipids in large VLDL	-0.052	-0.081 to -0.022	-0.054	-0.083 to -0.025
Total lipids in medium VLDL	-0.039	-0.069 to -0.010	-0.041	-0.070 to -0.012
Total lipids in IDL	-0.039	-0.070 to -0.009	-0.040	-0.070 to -0.009
Total lipids in large LDL	-0.045	-0.075 to -0.014	-0.045	-0.075 to -0.014
Total lipids in medium LDL	-0.043	-0.074 to -0.013	-0.044	-0.074 to -0.013
Total lipids in small LDL	-0.038	-0.069 to -0.007	-0.039	-0.070 to -0.008
Total lipids in small HDL	-0.054	-0.084 to -0.024	-0.048	-0.078 to -0.019
**Cholesterol**				
Cholesterol in VLDL	-0.039	-0.069 to -0.009	-0.038	-0.068 to -0.008
Remnant cholesterol (non-HDL, non-LDL -cholesterol)	-0.043	-0.073 to -0.012	-0.042	-0.073 to -0.012
Free cholesterol	-0.053	-0.083 to -0.023	-0.057	-0.087 to -0.027
**Glycerides and other Phospholipids**				
Serum total TG	-0.036	-0.066 to -0.006	-0.038	-0.068 to -0.009
TG in VLDL	-0.033	-0.062 to -0.004	-0.035	-0.064 to -0.006
TG in LDL	-0.029	-0.059 to 0.001	-0.032	-0.061 to -0.002
TG in HDL	-0.061	-0.091 to -0.032	-0.062	-0.092 to -0.032
Sphingomyelins	-0.081	-0.111 to -0.052	-0.081	-0.110 to -0.052
**Fatty Acids (FA)**				
Saturated FA	-0.056	-0.086 to -0.026	-0.057	-0.087 to -0.027
Monounsaturated FA	-0.069	-0.099 to -0.040	-0.070	-0.099 to -0.040
Polyunsaturated FA	0.075	0.045 to 0.105	0.077	0.046 to 0.107

Omega-3 FA	0.136	0.106 to	0.166	0.144	0.114 to	0.174	
docosahexaenoic acid	0.174	0.144 to	0.203	0.182	0.152 to	0.211	
Omega-6 FA	0.053	0.023 to	0.083	0.053	0.023 to	0.083	
linoleic acid	0.076	0.045 to	0.106	0.075	0.044 to	0.105	
Conjugated linoleic acid	-0.198	-0.228 to	-0.168	-0.198	-0.228 to	-0.169	[illegible]
Estimated degree of unsaturation	0.208	0.179 to	0.236	0.214	0.186 to	0.242	
**Fatty acids ratios, relative to total fatty acids**							
Ratio of saturated FA to total FA (%)	-0.162	-0.192 to	-0.132	-0.165	-0.195 to	-0.135	[illegible]
Ratio of monounsaturated FA to total FA (%)	-0.124	-0.153 to	-0.095	-0.125	-0.154 to	-0.096	[illegible]
Ratio of polyunsaturated FA to total FA (%)	0.193	0.164 to	0.221	0.195	0.167 to	0.224	
Ratio of omega-3 FA to total FA (%)	0.186	0.157 to	0.216	0.196	0.167 to	0.225	
Ratio of docosahexaenoic acid to total FA (%)	0.219	0.190 to	0.247	0.214	0.186 to	0.242	
Ratio of omega-6 FA to total FA (%)	0.137	0.108 to	0.166	0.137	0.107 to	0.166	
Ratio of linoleic acid to total FA (%)	0.146	0.116 to	0.175	0.144	0.115 to	0.174	
Ratio of conjugated linoleic acid to total FA (%)	-0.226	-0.256 to	-0.196	-0.226	-0.256 to	-0.197	[illegible]

Models adjusted for age, sex, total energy intake, ethnicity, smoking habits, physical activity, type 2 diabetes, diastolic and systolic blood pressure, use of antihypertensive drugs and use of lipid-lowering drugs. Results were expressed as linear regression coefficients accompanied with their 95 % confidence interval

Supplementary Table G: Metabolite concentrations in YFS participants – Replication analyses

	N	Mean	sd
**Amino Acids**			
Isoleucine (mmol/L)	1678	0.24	0.03
Leucine (mmol/L)	1678	0.30	0.03
Phenylalanine (mmol/L)	1678	0.30	0.02
**Glycolysis related metabolites**			
Glycerol(mmol/L)	1625	0.32	0.05
**Inflammation**			
Glycoprotein acetyls(mmol/L)	1678	1.29	0.11
**Size of lipoprotein particles (Mean diameters)**			
VLDL particle size (nm)	1679	6.01	0.11
HDL particle size(nm)	1679	3.16	0.04
**Total lipid concentrations in lipoprotein subclasses**			
Total lipids in chylomicrons and extremely large VLDL (mmol/L)	1679	0.14	0.08
Total lipids in very large VLDL (mmol/L)	1679	0.20	0.14
Total lipids in large VLDL (mmol/L)	1679	0.43	0.24
Total lipids in medium VLDL (mmol/L)	1679	0.72	0.22
Total lipids in IDL (mmol/L)	1679	1.15	0.14
Total lipids in large LDL (mmol/L)	1679	1.23	0.16
Total lipids in medium LDL (mmol/L)	1679	0.92	0.13
Total lipids in small LDL (mmol/L)	1679	0.73	0.10
Total lipids in small HDL (mmol/L)	1679	1.10	0.08
**Cholesterol**			
Cholesterol in VLDL(mmol/L)	1679	0.88	0.16
Remnant cholesterol (non-HDL, non-LDL -cholesterol) (mmol/L)	1679	1.27	0.18
Free cholesterol(mmol/L)	1677	1.27	0.12
**Glycerides and other Phospholipids**			
Serum total TG (mmol/L)	1679	1.10	0.28
TG in VLDL (mmol/L)	1679	0.86	0.28
TG in LDL (mmol/L)	1679	0.43	0.08
TG in HDL (mmol/L)	1679	0.39	0.06
Sphingomyelins (mmol/L)	1677	0.74	0.07
**Fatty Acids (FA)**			
Saturated FA (mmol/L)	1677	2.25	0.26
Monounsaturated FA (mmol/L)	1677	1.91	0.28
Polyunsaturated FA (mmol/L)	1677	2.18	0.21
Omega-3 FA (mmol/L)	1677	0.76	0.12
docosahexaenoic acid (mmol/L)	1677	0.49	0.07
Omega-6 FA (mmol/L)	1677	2.04	0.19
linoleic acid (mmol/L)	1677	1.84	0.17
**Fatty acids ratios, relative to total fatty acids**			

Estimated degree of unsaturation	1677	1.07	0.03
Ratio of saturated FA to total FA (%)	1677	6.12	0.16
Ratio of monounsaturated FA to total FA (%)	1677	5.19	0.27
Ratio of polyunsaturated FA to total FA (%)	1677	5.95	0.28
Ratio of omega-3 FA to total FA (%)	1677	2.07	0.24
Ratio of docosahexaenoic acid to total FA (%)	1677	1.33	0.15
Ratio of omega-6 FA to total FA (%)	1677	5.58	0.27
Ratio of linoleic acid to total FA (%)	1677	5.01	0.29

Supplementary Table H: Estimates of the multivariable-adjusted associations between AHEI z-score and metabolites from meta-analysis of the Whitehall II study and in Young Finns Study

	Beta	95% CI	p
**Amino Acids**			
Isoleucine	-0.030	-0.053 to -0.007	0.012
Leucine	-0.042	-0.065 to -0.019	<0.0001
Phenylalanine	-0.066	-0.092 to -0.040	<0.0001
**Glycolysis related metabolites**			
Glycerol	-0.035	-0.061 to -0.010	0.007
**Inflammation**			
Glycoprotein acetyls	-0.031	-0.056 to -0.006	0.02
**Size of lipoprotein particles (Mean diameters)**			
VLDL particle size	-0.037	-0.062 to -0.013	0.003
HDL particle size	0.024	0..001 to 0.048	0.044
**Total lipid concentrations in lipoprotein subclasses**			
Total lipids in chylomicrons and extremely large VLDL	-0.064	-0.089 to -0.039	<0.0001
Total lipids in very large VLDL	-0.051	-0.076 to -0.027	<0.0001
Total lipids in large VLDL	-0.039	-0.064 to -0.014	0.002
Total lipids in medium VLDL	-0.028	-0.052 to -0.003	0.03
Total lipids in small VLDL	-0.010	-0.035 to 0.015	0.428
Total lipids in IDL	-0.038	-0.064 to -0.012	0.004
Total lipids in large LDL	-0.042	-0.068 to -0.016	0.002
Total lipids in medium LDL	-0.040	-0.066 to -0.014	0.003
Total lipids in small LDL	-0.037	-0.063 to -0.011	0.006
Total lipids in small HDL	-0.041	-0.067 to -0.015	0.002
**Cholesterol**			
Cholesterol in VLDL	-0.029	-0.054 to -0.003	0.027
Remnant cholesterol (non-HDL, non-LDL -cholesterol)	-0.037	-0.062 to -0.011	0.005
Free cholesterol	-0.051	-0.076 to -0.025	<0.0001
**Glycerides and other Phospholipids**			
Serum total TG	-0.027	0.051 to -0.002	0.04
TG in VLDL	-0.023	-0.048 to 0.002	0.07
TG in LDL	-0.026	-0.052 to -0.001	0.04
TG in HDL	-0.050	-0.076 to -0.025	<0.0001

Sphingomyelins	-0.068	-0.093 to -0.043	<0.0001
**Fatty Acids (FA)**			
Saturated FA	-0.050	-0.075 to -0.025	<0.0001
Monounsaturated FA	-0.058	-0.083 to -0.033	<0.0001
Polyunsaturated FA	0.064	0.039 to 0.090	<0.0001
Omega-3 FA	0.130	0.104 to 0.155	<0.0001
docosahexaenoic acid	0.158	0.133 to 0.183	<0.0001
Omega-6 FA	0.043	0.017 to 0.068	0.001
linoleic acid	0.058	0.032 to 0.084	<0.0001
Conjugated linoleic acid	0.189	0.164 to 0.213	<0.0001
**Fatty acids ratios, relative to total fatty acids**			
Estimated degree of unsaturation	0.189	0.164 0.213	<0.0001
Ratio of saturated FA to total FA (%)	-0.146	-0.172 -0.120	<0.0001
Ratio of monounsaturated FA to total FA (%)	-0.101	-0.125 -0.076	<0.0001
Ratio of polyunsaturated FA to total FA (%)	0.167	0.142 0.191	<0.0001
Ratio of omega-3 FA to total FA (%)	0.179	0.154 0.204	<0.0001
Ratio of docosahexaenoic acid to total FA (%)	0.201	0.177 0.226	<0.0001
Ratio of omega-6 FA to total FA (%)	0.114	0.089 0.139	<0.0001
Ratio of linoleic acid to total FA (%)	0.117	0.091 0.142	<0.0001

Supplementary Table I: Construction of AHEI scores

Components		Criteria for min. scores	Criteria for max. scores	Possible score range	Scores in the WII participants (n=4824)		Scores in the YFS participants (n=1716)	
					Mean score (SD)	Median	Mean score (SD)	Median
Vegetable (serving /day)		0	5	0-10	6.5 (2.4)	6	7.3 (2.5)	8
Fruit (serving /day)		0	4	0-10	6.7 (2.8)	7	4.1 (2.6)	4
Nuts and Soy (serving /day)		0	1	0-10	1.9 (2.5)	1	1.7 (1.6)	1
Ratio of white to red meat		0	4	0-10	3.3 (2.7)	2	2.1 (1.7)	2
Trans Fat (% of energy)		≥4	≤0.5	0-10	9.6 (0.7)	10	9.9 (0.3)	10
Total Fiber (gram/day)		0	24	0-10	8.9 (1.7)	10	8.3 (2.0)	9
Ratio of PUFA to SFA		≤0.1	≥1	0-10	4.9 (2.2)	5	4.2 (1.2)	4
Duration of multivitamin Use		<5 year	≥5 year	2.5-7.5	3.8 (2.2)	2.5	4.7 (2.5)	2.5
Alcohol serving/day	Men	>3.5	1.5-2.5	5.5 (4.0) †	5.5 (4.0) †	4†	4.0 (3.9)	4
Alcohol serving/day	Women	>2.5	0.5-1.5					
Total Score				2.5-87.5	50.7 (9.8)	50.5	46.3 (8.0)	46.5

Abbreviation: AHEI, the alternative healthy eating index; WII, Whitehall II ; YFS, the Cardiovascular Risk in Young Finns Study ; PUFA, Polyunsaturated fatty acids; SAF, saturated fatty acids.

*Each AHEI component contributed from 0 to 10 points to the total AHEI score, except the multivitamin component which was dichotomous and contributing either 2.5 points (for non-use) or 7.5 points (for use) A score of 10 indicates that the recommendations were fully met, whereas a score of 0 represents the least healthy dietary behavior. Intermediate intakes were scored proportionately between 0 and 10.

† Median score for men and women combined. Nondrinkers received a score of 2.5

Supplementary Table J : Construction of AHEI 2010 scores

**Components**		**Criteria for min. scores**	**Criteria for max. scores**	**Scores in the WII participants (n=**4824)	
				Mean (sd)	Median
Vegetable (serving /day)		0	≥5	6.5 (2.4)	6
Fruit (serving /day)		0	≥4	5.8 (2.8)	6
Whole grains (serving /day)	Men	0	5	3.3 (2.6)	2
	Women	0	6		
Soda and fruit juice (serving /day)		≥1	0	3.4 (3.6)	2
Nuts and legumes (serving /day)		0	1	2.5 (2.8)	2
Processed /Red Meat		≥1.5	0	5.1 (2.8)	5
Trans Fat (% of energy)		≥4	≤0.5	5.0 (3.1)	5
Long-chain (n-3) fats, *mg/d*		0	250	8.1 (2.6)	10
PUFA*, *% of energy*		≤2	≥10	3.7 (2.8)	3
Sodium, *mg/d*		Highest decile	Lowest decile	5.0 (3.2)	5
Alcohol serving/day	Men	>3.5	≤ 1.5	5.5 (4.0)	3
	Women	>2.5	≤ 0.5		
Total Score				53.8 (10.0)	

* PUFA (Polyunsaturated fatty acids) does not include n-3 PUFA.

Each AHEI component contributed from 0 to 10 points to the total AHEI-2010 score. A score of 10 indicates that the recommendations were fully met, whereas a score of 0 represents the least healthy dietary behavior. Intermediate intakes were scored proportionately between 0 and 10. All the component scores are summed to obtain the total AHEI-2010 score
